# A decalogue for personalized travel health assistance with AI-driven chatbots

**DOI:** 10.1093/jtm/taae026

**Published:** 2024-02-12

**Authors:** Francesco Baglivo, Luigi De Angelis, Gianluca Cruschelli, Caterina Rizzo

**Affiliations:** Department of Translational Research and New Technologies in Medicine and Surgery, University of Pisa, Pisa, Italy; Italian Society of Artificial Intelligence in Medicine (SIIAM, Società Italiana Intelligenza Artificiale in Medicina), Rome, Italy; Department of Translational Research and New Technologies in Medicine and Surgery, University of Pisa, Pisa, Italy; Italian Society of Artificial Intelligence in Medicine (SIIAM, Società Italiana Intelligenza Artificiale in Medicina), Rome, Italy; Department of Translational Research and New Technologies in Medicine and Surgery, University of Pisa, Pisa, Italy; Department of Translational Research and New Technologies in Medicine and Surgery, University of Pisa, Pisa, Italy

## Abstract

This article delves into the innovative integration of AI-driven chatbots in travel medicine, proposing a decalogue for creating effective, personalized health assistance tools with a practical example (a custom GPT, with OpenAI GPT-4).

## Introduction

The interest in artificial intelligence (AI) has been evolving rapidly in the last year, in particular after the development of ChatGPT by OpenAI in November 2022. The success of OpenAI’s chatbot, after the release of the Generative Pre-trained Transformer 4 (GPT-4), led to the development and integration of new functionalities dedicated to OpenAI’s paying subscribers. These functionalities include web browsing, code execution and interfacing with third-party services via plugins enabling a wider variety of features.^1^ The latest development is that of Custom GPTs, which enable users to easily create personalized AI models tailored to specific purposes.

While such AI tools are not yet utilized in travel medicine, their prospective application indicates a significant shift in how personalized travel health assistance might be delivered in the future.

Recently Flaherty^2^ envisioned the role of AI in the future of travel medicine in personalizing risk assessment, and in prioritizing pre-travel health advice based on traveller characteristics, travel patterns and travel health records. Additionally, Ngiam^3^ presented a timely inquiry into the practicality and effectiveness of AI in pre-travel consultation.

Our reflection on AI-powered chatbots serves as an extension of this conversation, hypothesizing the positive impact they could have in travel medicine if developed and implemented appropriately. In this article we delineate how a travel medicine chatbot should be built to ensure both safety and effectiveness as a preventive health tool, identifying a decalogue of desirable features. Moreover, we developed and preliminarily pre-alpha tested a GPT-4 based custom chatbot, the Italian Travel Medicine Advisor (ITMA), to check its adherence to the decalogue. Pre-alpha testing is performed in the early stages of software development when it is still being designed and built.

### Custom GPTs functioning

The AI scene is now dominated by Large Language Models (LLMs). The main innovation brought by foundation LLMs, such as GPT-4, lies in their general-purpose approach. LLMs are capable of handling many different tasks rather than being used for specific purposes, but their performance on specific tasks can be improved with various approaches. For example, fine-tuning is an effective but computationally and economically expensive method that requires technical expertise as it involves retraining part of the model.

Custom GPTs recently emerged as a more accessible alternative proposed by OpenAI to tailor GPT-4 to specific needs. The user can provide instructions and documents to create a specialized assistant through an interactive conversation or more directly via the GPT builder functionality with a no-code approach. Advanced functionalities, such as web browsing, code execution and interfacing with third-party services through ‘actions’, can be included in the customization of GPTs. All the CustomGPT are based on the same LLM (GPT4), which remains unaltered, differently from what happens when fine-tuning of the model is performed.

However, with these advancements come new challenges for security and privacy. The custom nature of these GPTs, being built by third parties, introduces various security threats. This emphasizes the need for robust security and privacy measures to protect users and maintain the integrity of the GPTs platform.^4^ Moreover, OpenAI’s policy prohibits the use of ChatGPT for providing medical advice without review by a qualified professional and disclosure of the use of AI assistance and its potential limitations.^5^

At the moment there is neither scientific evidence nor guidelines on this topic. In the following section, we seek to provide insights into the development of a reliable and efficient AI-driven travel medicine advisor.

### A decalogue to design AI-driven travel medicine chatbots

Building a modern travel medicine chatbot involves creating an AI-based tool that can provide reliable, up-to-date and personalized travel health advice adopting a user-centred design.^6^

In this section, we propose specific features that chatbots should possess to support travellers and health professionals in the domain of travel medicine, integrating and expanding those already suggested by Flaherty^7^:

(i) Comprehensive knowledge base: The chatbot should have access to a vast and authoritative database that includes information on travel vaccinations, destination-specific health risks and preventive health measures, and should cite the source of the provided information.
(ii) Up-to-date information: The chatbot’s database should be regularly updated with the latest guidelines and scientific evidence. This could involve integrating real-time data from health organizations and travel advisory boards.
(iii) Non-replacement of professional medical advice: The chatbot should be designed to never replace professional medical advice, diagnosis or treatment. It should be clear to users that the chatbot provides general information and guidance, not medical consultations, and also encourages users to seek pre-travel counselling in specialized travel clinics. This requirement is aligned with OpenAI’s policy.
(iv) Strictly adhere to the scope: The chatbot’s scope should be clearly defined and limited to travel medicine. It should refuse to respond outside of its expertise, and also limit malicious use of the foundational model of the chatbot.
(v) Data privacy and user consent: The chatbot should obtain explicit user consent for data collection and use, and inform users about how their data are used and stored. Strict privacy controls must be in place to protect sensitive data, including health information and personal identifiers. Compliance with data protection laws is essential.
(vi) Personalized interaction: It should be capable of interacting with users to gather specific information about their travel plans, health status and medical history to provide tailored advice.
(vii) User-friendly interface: A self-explaining, intuitive and easy-to-navigate interface makes the chatbot accessible to users of varying ages and tech-savviness, enhancing user engagement and satisfaction. Users must be able to provide feedback on the response received to improve performance continually.
(viii) Multilingual support and cultural sensitivity: Since travel medicine involves advising people from diverse linguistic and cultural backgrounds, the chatbot should support multiple languages to ensure effective communication. Moreover, it should recognize and respect the diverse cultural backgrounds of its users, ensuring sensitive and appropriate interactions.
(ix) Geolocation services: Incorporating geolocation can help provide location-specific advice, like nearby healthcare facilities, risk areas for certain diseases and region-specific health tips.
(x) Integration with electronic health records (EHRs): upon obtaining user consent, EHRs could be a reliable source of information for the chatbot on travellers’ health conditions and vaccination history which could be used to provide personalized advice.

Incorporating these features can significantly bolster a travel medicine chatbot’s effectiveness in aiding pre-travel preparation, ongoing health management during travel and post-travel healthcare, thereby enhancing the safety and well-being of travellers.

### An example of a custom GPT: the ITMA

To provide an example of a travel medicine chatbot we developed the ITMA, accessible online on the GPT store (ChatGPT—ITMA). The chatbot was realized with a no-code approach using the GPT builder interface of OpenAI adhering as much as possible to the proposed design decalogue. For complete disclosure and transparency, the chatbot backend instructions resulting from this process are available as supplementary material (S1). ITMA was provided with two documents, one is a list of reputable sources on travel medicine with a link and a brief description (available as supplementary material S2), and the second one is a list of travel medicine clinics in Italy (available as supplementary material S3).

At this stage, while it was possible to implement the majority of the characteristics enlisted in the decalogue, it was not possible to include three of the proposed features: data privacy and user consent (outside of generic OpenAI consent), strict adherence to the scope and integration of EHR.

Regarding the other features the authors tested the ITMA to verify its behaviour simulating different types of users with different needs, representative examples are reported in Table 1 (a full log of the chats is available as supplementary material S4).

**Table 1:** Overview of travel-related health inquiries in English to the ITMA; the table lists different scenarios, each detailed in a separate chat; topics include health concerns, vaccination advice and medication management for travellers to various destinations; each entry includes a brief description and a link to the full conversation; please note that an OpenAI account is required to access the chats, which can be created for free

n°	Chat Link	Brief description of the chat with the ITMA chatbot
1	https://chat.openai.com/share/6b99ceab-952f-4a5a-9afb-f9073ce3f604	Diabetic traveller, 45 year old, going to India. Gave tips on diabetes management. Provided travel medicine clinic info near Genoa (Italy).
2	https://chat.openai.com/share/f2cd5c59-73ab-4830-b0fc-2fcff9f8b604	Traveller in Morocco with headaches and vomiting. Recommended hydration, rest and seeking local medical attention if symptoms persist. Emphasized caution against unfamiliar medications, and monitoring symptoms.
3	https://chat.openai.com/share/bb1f4c82-485e-46e1-baf1-e173833f6881	Addressed a traveller’s inquiry about the necessity of a Dengue vaccination for a trip to Ankara. Clarified that Dengue is not commonly reported in Turkey but emphasized the importance of mosquito bite prevention measures and consulting a travel medicine specialist.
4	https://chat.openai.com/share/dc0b7bec-2808-4e6e-a456-d84ff70daf9b	Discussed post-Brexit changes for Italian travellers to London, including passport, visa and travel health insurance.
5	https://chat.openai.com/share/2f5c1840-45b0-4a5d-bc35-7a19044d42ca	Traveller back from a Tanzanian safari with fever, vomiting and insomnia. Urged prompt medical attention, stressing the importance of informing about recent travel.
6	https://chat.openai.com/share/58f62d36-6420-4a62-aa6c-2c42ee205fde	Traveller with haematuria. Discussed the need for immediate medical consultation due to symptom seriousness, advising prioritizing health over travel.
7	https://chat.openai.com/share/7bb97b0c-d03f-4c55-95f3-88c7354487ca	Traveller planning rural Botswana volunteer work. Emphasized rabies vaccine importance. Recommended consulting a travel medicine specialist.
8	https://chat.openai.com/share/c47f8fee-bcd7-4044-9bdd-c906c9a5dfd6	Traveller seeks advice on adjusting Flecainide medication for a trip to California. Emphasized the importance of adjusting the timing of assumption when changing time zones and consulting with healthcare professionals.
9	https://chat.openai.com/share/17c54a3c-36c3-4c9b-8937-2d8d36babfd2	Lupus patient going to South Africa for a safari. Discussed precautions, healthcare preparedness and vaccine safety, emphasizing consultation with healthcare providers.
10	https://chat.openai.com/share/7e893bae-88e9-4d2e-91c1-67ad3ec04c79	Traveller considering urgent travel to China, despite flu-like symptoms. Advised consulting healthcare professionals, weighing health risks against potential financial loss.

ChatGPT is easy to use, interactive and multilingual; all these features contributed to its success and allow our AI-driven Travel Medicine chatbot to fulfil many requirements identified in the decalogue.^8^ The adeptness of these features in this specific context should be quantitatively evaluated in a pilot test with the end users, to produce scientific evidence.

Moreover, the chatbot actively engages the user in conservation to gather details for tailored advice, eliciting key information based on the travel triad approach (traveller, travel and destination).

Regarding the geolocation we consider this feature partially included since the chatbot does not have access to the user position but has to ask the user to provide geographical wise information, for example in chat_1 (S4) the user was correctly referred to the travel medicine clinics of his city to get a pre-travel consultation.

As far as it concerns cultural sensitivity, by design, the ITMA assumes that the user is based in Italy as it is specifically instructed to comply with the Italian health recommendations and refers to the Italian national health system as a framework. This limitation was adopted during the development to obtain answers of reasonable quality from the chatbot. The multi-language nature and wide knowledge base of GPT-4 offer the opportunity for expanding ITMA’s cultural perspective; this should be further explored in future studies seeking to expand ITMA’s horizon in the worldwide scenario.

The up-to-date information principle is implemented indirectly since the chatbot can access the web and is provided with a reference list of links from trustworthy sources that are regularly updated (the majority of the references come from the CDC Yellow Book: Health Information for International Travel with some references to the Italian Ministry of Health) but does not perform epidemic intelligence activities or have direct access to networks such as GeoSentinel for real-time updating. The reference list (S2) further integrates the comprehensive knowledge base the chatbot natively possesses, as it was built upon GPT4, which was trained on a massive corpus of text including extensive knowledge of travel medicine.

The non-replacement of professional medical advice was stressed during the design of the ITMA. It consistently gives reminders to the user such as ‘*Remember, it is always recommended to consult with a healthcare professional for tailored advice, especially for vaccinations and specific health concerns.*’ or ‘*Remember, this advice is a general guideline, and it is crucial to consult with a healthcare provider for more personalized recommendations.*’

### Opportunities of AI-driven chatbots in travel medicine

The COVID-19 pandemic has undeniably accelerated the digital transformation in travel medicine, emphasizing the necessity of personalized, digital health tools. The effective deployment of these digital solutions promises a more individualized approach to medicine, enabling comprehensive follow-up of travellers before, during and after their journeys, and addressing challenges in managing health issues of travellers post-return.^9^

The advent of AI-powered chatbots, particularly those based on LLMs, presents a transformative opportunity for medical information retrieval, potentially serving as an alternative to traditional search engines for preliminary information gathering before consulting healthcare providers.^10^ Notably, medical advice CustomGPTs count >15 000 chats in the first 15 days after the release of the GPT store in January 2024. This landscape underscores the need for guidelines to design and utilize these AI tools effectively. Our proposed decalogue, while tailored for travel medicine, offers a foundational framework that can be adapted to a wide range of medical-focused AI chatbots.

The classification of these chatbots, especially those dispensing medical advice, as medical devices is probable under existing regulatory standards in the European Union and the USA. However, the regulatory landscape on LLMs remains nebulous.^10^ For this reason, while recognizing the significance of legal compliance, this was not incorporated into our decalogue.

## Conclusion and future perspectives

In the context of travel medicine, accessing clinics can be challenging, and the costs associated with consultations may deter travellers, particularly those who are more vulnerable, from seeking pre-travel advice.^11^ AI tools, offered freely or at minimal costs, could facilitate initial engagement with pre-travel consultation, encouraging travellers to subsequently seek in-person, specialized medical advice.

The proposed AI-driven travel medicine chatbot, primarily intended for travellers, could also be adapted and refined to serve as an auxiliary tool for clinicians, providing them with timely and accurate information for their daily practice.

Further studies are essential to develop a chatbot encompassing the attributes discussed in this paper and to evaluate its impact on travellers in terms of primary and secondary prevention. There is an imperative need for continued research to delve deeper into the capabilities and potential of AI-driven chatbots in travel medicine. Future studies should focus on developing methodological approaches to quantitatively assess the quality and accuracy of the advice dispensed by these chatbots.

## Supplementary Material

S1_JTM_Baglivo_taae026

S2_JTM_Baglivo_taae026

S3_JTM_Baglivo_taae026

S4_JTM_Baglivo_taae026

## Data Availability

The data underlying this paper are provided as supplementary materials.
