# Supplementary material for: A decalogue for personalized travel health assistance with AI-driven chatbots
Source: J Travel Med. 2024 Feb 12;31(4):taae026. doi: 10.1093/jtm/taae026 (PMC11149716; doi:10.1093/jtm/taae026)
Supplement: S2_JTM_Baglivo_taae026 [file s2_jtm_baglivo_taae026.docx]

Authoritative Sources for Travel Medicine Information

# Introduction

This document is compiled to serve as a resource for the Italian Travel Medicine Advisor GPT, aimed at providing accurate, reliable, and up-to-date travel health and medicine information. The websites listed herein have been carefully selected for their authority, credibility, and relevance to travel medicine by the authors. This guide will aid the GPT in sourcing information to address queries related to pre-travel, during-travel, and post-travel health concerns.

# Purpose of This Document

The primary purpose of this document is to:

- Provide the GPT with a comprehensive list of trusted sources for travel health information.

- Ensure that the advice and information relayed by the GPT are based on credible and current resources.

- Facilitate the GPT’s ability to deliver well-informed and accurate health recommendations to users.

# Explanation of Selected Websites

Each website listed in this document has been chosen based on several criteria:

1. Authority and Accuracy: Websites are official sources like health organizations, medical institutions, or government health departments known for their accuracy and authoritative information.

2. Relevance to Travel Medicine: The content of these websites specifically relates to travel health, covering aspects such as vaccinations, health advisories, disease prevention, and global health alerts.

3. Timeliness: The selected sources are known for providing up-to-date information, crucial for travel medicine advice where recommendations can change rapidly.

4. Accessibility and Clarity: Websites that present information in a clear, comprehensible manner, making them accessible to a diverse audience.

# How the GPT Will Utilize These Sources

- The GPT will reference these websites to answer specific user queries about travel-related health issues.

- In cases of detailed or complex medical queries, the GPT will direct users to these websites for further information.

- The GPT will use this information to stay updated on the latest travel health advisories and recommendations.

# Regular Review and Update

- This document will be reviewed and updated periodically to ensure that the GPT has access to the most current and relevant information.

- Any changes in the listed websites (such as updates or new sources) will be reflected in subsequent versions of this document.

**Current version: 16/01/2024**

# Table of Authoritative Sources for Travel Medicine Information

| **n°** | **Website Name** | **URL** | **Description** | **Relevance to Travel Medicine** |
| --- | --- | --- | --- | --- |
| 1 | CDC - Travelers' Health: Immunocompromised Travelers | <https://wwwnc.cdc.gov/travel/yellowbook/2024/additional-considerations/immunocompromised-travelers> | This section of the CDC's Yellow Book provides comprehensive guidelines and advice for immunocompromised travelers, covering vaccinations, health precautions, and travel planning. | Offers specific and detailed medical advice tailored for travelers with compromised immune systems, a key demographic in travel medicine. |
| 2 | CDC - Travelers with Disabilities | <https://wwwnc.cdc.gov/travel/yellowbook/2024/additional-considerations/travelers-with-disabilities> | Provides information on planning and preparing for travel, considering the unique needs of travelers with disabilities. | Addresses accessibility and health management for travelers with disabilities. |
| 3 | CDC - Travelers with Chronic Illnesses | <https://wwwnc.cdc.gov/travel/yellowbook/2024/additional-considerations/travelers-with-chronic-illnesses> | Offers guidelines for travelers with chronic illnesses, focusing on medication management and healthcare access while traveling. | Essential for advising travelers managing ongoing health conditions. |
| 4 | CDC - Highly Allergic Travelers | <https://wwwnc.cdc.gov/travel/yellowbook/2024/additional-considerations/highly-allergic-travelers> | Discusses precautions and preparations for travelers with severe allergies, including food and medication allergies. | Critical for ensuring the safety of travelers with high allergy risks. |
| 5 | CDC - Substance Use | <https://wwwnc.cdc.gov/travel/yellowbook/2024/additional-considerations/substance-use> | Information on considerations for travelers with substance use concerns, including legal and health-related aspects. | Important to address the needs and risks associated with substance use while traveling. |
| 6 | CDC - Sun Exposure | <https://wwwnc.cdc.gov/travel/yellowbook/2024/environmental-hazards-risks/sun-exposure> | Guidelines on protecting against harmful sun exposure, including sunscreen use and protective clothing. | Vital for travelers to sunny destinations; skin protection. |
| 7 | CDC - Extremes of Temperature | <https://wwwnc.cdc.gov/travel/yellowbook/2024/environmental-hazards-risks/extremes-of-temperature> | Advice on dealing with extreme heat and cold during travel, emphasizing prevention and response. | Essential for travel to destinations with extreme temperature conditions. |
| 8 | CDC - Diving | <https://wwwnc.cdc.gov/travel/yellowbook/2024/environmental-hazards-risks/diving> | Safety guidelines and health considerations for recreational diving activities. | Important for travelers engaging in underwater activities. |
| 9 | CDC - High Elevation Travel | <https://wwwnc.cdc.gov/travel/yellowbook/2024/environmental-hazards-risks/high-elevation-travel-and-altitude-illness> | Information on altitude illness prevention and management for travelers to high-elevation areas. | Critical for mountain and high-altitude destination travelers. |
| 10 | CDC - Mosquitoes, Ticks, Arthropods | <https://wwwnc.cdc.gov/travel/yellowbook/2024/environmental-hazards-risks/mosquitoes-ticks-and-other-arthropods> | Preventive measures against diseases transmitted by mosquitoes, ticks, and other arthropods. | Essential for travelers to areas with vector-borne diseases. |
| 11 | CDC - Zoonotic Exposures | <https://wwwnc.cdc.gov/travel/yellowbook/2024/environmental-hazards-risks/zoonotic-exposures-bites-stings-scratches-and-other-hazards> | Information on avoiding zoonotic disease transmission through animal bites, stings, and scratches. | Important for interactions with wildlife and domestic animals. |
| 12 | CDC - Zoonoses: One Health Approach | <https://wwwnc.cdc.gov/travel/yellowbook/2024/environmental-hazards-risks/zoonoses-one-health-approach-> | Overview of the One Health approach to understanding and preventing zoonotic diseases. | Offers a holistic view of health and disease ecology in travel. |
| 13 | CDC - Food Poisoning from Marine Toxins | <https://wwwnc.cdc.gov/travel/yellowbook/2024/environmental-hazards-risks/food-poisoning-from-marine-toxins> | Guidelines on avoiding food poisoning from marine toxins, focusing on seafood safety. | Crucial for travelers consuming seafood in coastal regions. |
| 14 | CDC - Safety and Security Overseas | <https://wwwnc.cdc.gov/travel/yellowbook/2024/environmental-hazards-risks/safety-and-security-overseas> | Tips on staying safe and secure while traveling abroad, including personal security measures. | Important for overall traveler safety in various international contexts. |
| 15 | CDC - Injury and Trauma | <https://wwwnc.cdc.gov/travel/yellowbook/2024/environmental-hazards-risks/injury-and-trauma> | Information on preventing and managing injuries and trauma while traveling. | Addresses a range of potential physical injuries during travel. |
| 16 | CDC - Poisonings, Envenomations, Toxic Exposures | <https://wwwnc.cdc.gov/travel/yellowbook/2024/environmental-hazards-risks/poisonings-envenomations-and-toxic-exposures> | Precautions and treatments for poisonings, envenomations, and toxic exposures during travel. | Critical for handling encounters with poisonous or venomous creatures and substances. |
| 17 | CDC - Vaccine-Preventable Bacterial Diseases | <https://wwwnc.cdc.gov/travel/yellowbook/2024/infections-diseases/vaccine-preventable-diseases-bacterial> | Information on bacterial diseases preventable by vaccines, important for travel planning. | Essential for advising travelers on necessary vaccinations. |
| 18 | CDC - Anthrax | <https://wwwnc.cdc.gov/travel/yellowbook/2024/infections-diseases/anthrax> | Guidelines on anthrax, including risk factors and prevention strategies. | Important for travelers to areas where anthrax is a risk. |
| 19 | CDC - Bartonella Infections | <https://wwwnc.cdc.gov/travel/yellowbook/2024/infections-diseases/bartonella-infections> | Information on Bartonella infections, including transmission and prevention. | Relevant for understanding and preventing Bartonella infections during travel. |
| 20 | CDC - Brucellosis | <https://wwwnc.cdc.gov/travel/yellowbook/2024/infections-diseases/brucellosis> | Details on brucellosis, focusing on risks and preventive measures. | Crucial for travelers to regions where brucellosis is prevalent. |
| 21 | CDC - Campylobacteriosis | <https://wwwnc.cdc.gov/travel/yellowbook/2024/infections-diseases/campylobacteriosis> | Covers campylobacteriosis, including symptoms, treatment, and prevention. | Useful for addressing gastrointestinal issues in travelers. |
| 22 | CDC - Cholera | <https://wwwnc.cdc.gov/travel/yellowbook/2024/infections-diseases/cholera> | Comprehensive guide on cholera, including vaccination and preventive advice. | Important for travel to areas with known cholera risk. |
| 23 | CDC - Diphtheria | <https://wwwnc.cdc.gov/travel/yellowbook/2024/infections-diseases/diphtheria> | Information on diphtheria, including symptoms, transmission, and prevention. | Vital for travelers needing diphtheria vaccination advice. |
| 24 | CDC - Diarrheagenic E. Coli | <https://wwwnc.cdc.gov/travel/yellowbook/2024/infections-diseases/escherichia-coli-diarrheagenic> | Advice on prevention and management of diarrheagenic E. coli infections. | Crucial for managing traveler's diarrhea risks. |
| 25 | CDC - Helicobacter Pylori | <https://wwwnc.cdc.gov/travel/yellowbook/2024/infections-diseases/helicobacter-pylori> | Overview of Helicobacter pylori, including transmission and prevention. | Relevant for travelers concerned about gastric issues. |
| 26 | CDC - Legionnaires’ Disease | <https://wwwnc.cdc.gov/travel/yellowbook/2024/infections-diseases/legionnaires-disease-and-pontiac-fever> | Information on Legionnaires’ disease and Pontiac fever, focusing on prevention. | Important for understanding respiratory risks in certain environments. |
| 27 | CDC - Leptospirosis | <https://wwwnc.cdc.gov/travel/yellowbook/2024/infections-diseases/leptospirosis> | Guidelines on leptospirosis, including risks and preventive strategies. | Essential for travelers to areas with waterborne disease risks. |
| 28 | CDC - Lyme Disease | <https://wwwnc.cdc.gov/travel/yellowbook/2024/infections-diseases/lyme-disease> | Comprehensive advice on Lyme disease, its prevention, and areas of risk. | Crucial for travelers to regions with tick populations. |
| 29 | CDC - Melioidosis | <https://wwwnc.cdc.gov/travel/yellowbook/2024/infections-diseases/melioidosis> | Detailed information on melioidosis, including transmission and prevention. | Important for travelers to tropical and subtropical regions. |
| 30 | CDC - Meningococcal Disease | <https://wwwnc.cdc.gov/travel/yellowbook/2024/infections-diseases/meningococcal-disease> | Information on meningococcal disease, including vaccine recommendations. | Critical for travelers to areas with meningococcal disease risk. |
| 31 | CDC - Pertussis (Whooping Cough) | <https://wwwnc.cdc.gov/travel/yellowbook/2024/infections-diseases/pertussis-whooping-cough> | Guidelines on pertussis, focusing on vaccination and prevention for travelers. | Essential for preventing whooping cough, especially in areas where it's prevalent. |
| 32 | CDC - Plague | <https://wwwnc.cdc.gov/travel/yellowbook/2024/infections-diseases/plague> | Advice on avoiding plague risks and necessary precautions while traveling. | Important for travel to regions where plague is a risk. |
| 33 | CDC - Pneumococcal Disease | <https://wwwnc.cdc.gov/travel/yellowbook/2024/infections-diseases/pneumococcal-disease> | Comprehensive information on pneumococcal disease, including vaccines. | Vital for understanding pneumococcal vaccination needs for travelers. |
| 34 | CDC - Q Fever | <https://wwwnc.cdc.gov/travel/yellowbook/2024/infections-diseases/q-fever> | Overview of Q fever, its risks, and preventive measures for travelers. | Relevant for travelers to farming areas or those exposed to animals. |
| 35 | CDC - Rickettsial Diseases | <https://wwwnc.cdc.gov/travel/yellowbook/2024/infections-diseases/rickettsial-diseases> | Information on rickettsial diseases, including symptoms and prevention. | Important for travelers to areas with ticks and flea-borne diseases. |
| 36 | CDC - Salmonellosis (Nontyphoidal) | <https://wwwnc.cdc.gov/travel/yellowbook/2024/infections-diseases/salmonellosis-nontyphoidal> | Guidelines on nontyphoidal salmonellosis, including prevention and treatment. | Crucial for managing food and water safety on travels. |
| 37 | CDC - Shigellosis | <https://wwwnc.cdc.gov/travel/yellowbook/2024/infections-diseases/shigellosis> | Information on shigellosis and its prevention, especially for travelers. | Useful for addressing bacterial diarrhea risks in travelers. |
| 38 | CDC - Tetanus | <https://wwwnc.cdc.gov/travel/yellowbook/2024/infections-diseases/tetanus> | Comprehensive guide on tetanus, including vaccine advice for travelers. | Essential for travelers, especially those at risk of cuts or wounds. |
| 39 | CDC - Tuberculosis | <https://wwwnc.cdc.gov/travel/yellowbook/2024/infections-diseases/tuberculosis> | Details on tuberculosis risks and prevention for travelers. | Important for travel to areas with higher incidence of tuberculosis. |
| 40 | CDC - Testing for Mycobacterium Tuberculosis | <https://wwwnc.cdc.gov/travel/yellowbook/2024/infections-diseases/testing-travelers-for-mycobacterium-tuberculosis-infection> | Guidelines on testing for Mycobacterium tuberculosis infection in travelers. | Relevant for assessing tuberculosis risk in travelers returning from endemic areas. |
| 41 | CDC - Typhoid and Paratyphoid Fever | <https://wwwnc.cdc.gov/travel/yellowbook/2024/infections-diseases/typhoid-and-paratyphoid-fever> | Information on typhoid and paratyphoid fever, including vaccine recommendations. | Crucial for travelers to regions where typhoid fever is common. |
| 42 | CDC - Yersiniosis | <https://wwwnc.cdc.gov/travel/yellowbook/2024/infections-diseases/yersiniosis> | Overview of yersiniosis, its transmission, and preventive measures for travelers. | Useful for understanding the risks and prevention of yersiniosis while traveling. |
| 43 | CDC - Vaccine-Preventable Viral Diseases | <https://wwwnc.cdc.gov/travel/yellowbook/2024/infections-diseases/vaccine-preventable-diseases-viral> | Information on viral diseases preventable by vaccines, including travel vaccination guidelines. | Vital for advising travelers on necessary vaccinations for viral diseases. |
| 44 | CDC - B Virus | <https://wwwnc.cdc.gov/travel/yellowbook/2024/infections-diseases/b-virus> | Details about B virus, including transmission and prevention for travelers. | Important for travelers to areas with potential B virus exposure risks. |
| 45 | CDC - Chikungunya | <https://wwwnc.cdc.gov/travel/yellowbook/2024/infections-diseases/chikungunya> | Guidelines on chikungunya, focusing on prevention, symptoms, and areas of risk. | Relevant for travelers to regions where chikungunya is prevalent. |
| 46 | CDC - COVID-19 | <https://wwwnc.cdc.gov/travel/yellowbook/2024/infections-diseases/covid-19> | Comprehensive advice on COVID-19, including travel-related guidelines and health measures. | Critical for all travelers in the context of the ongoing global pandemic. |
| 47 | CDC - Dengue | <https://wwwnc.cdc.gov/travel/yellowbook/2024/infections-diseases/dengue> | Information on dengue fever, including prevention and endemic areas. | Essential for travelers to tropical and subtropical regions where dengue is common. |
| 48 | CDC - Hand, Foot, and Mouth Disease | <https://wwwnc.cdc.gov/travel/yellowbook/2024/infections-diseases/hand-foot-and-mouth-disease> | Overview of hand, foot, and mouth disease, with a focus on travel-related considerations. | Useful for understanding and preventing this common viral illness in travelers. |
| 49 | CDC - Henipavirus Infections | <https://wwwnc.cdc.gov/travel/yellowbook/2024/infections-diseases/henipavirus-infections> | Information on henipavirus infections, their transmission, and preventive measures. | Relevant for travelers to areas where henipavirus is a concern. |
| 50 | CDC - Hepatitis A | [Link](https://wwwnc.cdc.gov/travel/yellowbook/2024/infections-diseases/hepatitis-a) | Guidelines on hepatitis A, including vaccine advice and prevention tips. | Crucial for travelers, especially to regions with higher hepatitis A prevalence. |
| 51 | CDC - Hepatitis B | <https://wwwnc.cdc.gov/travel/yellowbook/2024/infections-diseases/hepatitis-b> | Comprehensive guide on hepatitis B, focusing on vaccination and travel risks. | Important for travelers to areas with high rates of hepatitis B. |
| 52 | CDC - Hepatitis C | <https://wwwnc.cdc.gov/travel/yellowbook/2024/infections-diseases/hepatitis-c> | Information about hepatitis C, including transmission risks and prevention for travelers. | Relevant for understanding the risks and prevention of hepatitis C during travel. |
| 53 | CDC - Hepatitis E | <https://wwwnc.cdc.gov/travel/yellowbook/2024/infections-diseases/hepatitis-e> | Overview of hepatitis E, including transmission and preventive advice for travelers. | Important for travel to regions where hepatitis E is endemic. |
| 54 | CDC - HIV | <https://wwwnc.cdc.gov/travel/yellowbook/2024/infections-diseases/hiv> | Guidelines on traveling with HIV, including medication management and healthcare access. | Essential for travelers managing HIV, offering advice on care and precautions. |
| 55 | CDC - Influenza | <https://wwwnc.cdc.gov/travel/yellowbook/2024/infections-diseases/influenza> | Information on influenza and its prevention, particularly in the context of travel. | Vital for travelers, especially during flu season and in areas with higher flu activity. |
| 56 | CDC - Japanese Encephalitis | <https://wwwnc.cdc.gov/travel/yellowbook/2024/infections-diseases/japanese-encephalitis> | Details on Japanese encephalitis, including vaccine recommendations. | Important for travelers to rural areas in Asia and the Western Pacific. |
| 57 | CDC - MERS (Middle East Respiratory Syndrome) | <https://wwwnc.cdc.gov/travel/yellowbook/2024/infections-diseases/mers> | Information on MERS, including transmission and preventive measures for travelers. | Important for travel to areas where MERS is present, particularly the Arabian Peninsula. |
| 58 | CDC - Mumps | <https://wwwnc.cdc.gov/travel/yellowbook/2024/infections-diseases/mumps> | Overview of mumps, focusing on vaccination and prevention strategies for travelers. | Relevant for travelers, considering outbreaks in various regions. |
| 59 | CDC - Norovirus | <https://wwwnc.cdc.gov/travel/yellowbook/2024/infections-diseases/norovirus> | Guidelines on norovirus prevention, commonly affecting travelers in various settings. | Crucial for managing gastroenteritis risks, especially on cruises and in resorts. |
| 60 | CDC - Poliomyelitis (Polio) | <https://wwwnc.cdc.gov/travel/yellowbook/2024/infections-diseases/poliomyelitis> | Information on polio, including vaccination recommendations for travelers. | Essential for travel to countries where polio is still a risk. |
| 61 | CDC - Rabies | <https://wwwnc.cdc.gov/travel/yellowbook/2024/infections-diseases/rabies> | Comprehensive advice on rabies risk and prevention for travelers. | Vital for travelers to areas with high risk of rabies in animals. |
| 62 | CDC - Rabies Immunization | <https://wwwnc.cdc.gov/travel/yellowbook/2024/infections-diseases/rabies-immunization> | Detailed guidelines on rabies immunization for travelers. | Important for understanding pre- and post-exposure rabies vaccination. |
| 63 | CDC - Rubella | <https://wwwnc.cdc.gov/travel/yellowbook/2024/infections-diseases/rubella> | Information on rubella, including vaccine advice for travelers. | Relevant for ensuring immunity against rubella, especially in outbreak regions. |
| 64 | CDC - Rubeola (Measles) | <https://wwwnc.cdc.gov/travel/yellowbook/2024/infections-diseases/rubeola-measles> | Overview of measles, focusing on vaccination and prevention for travelers. | Crucial for travel, given the global resurgence of measles. |
| 65 | CDC - Smallpox and Other Orthopoxvirus-Associated Infections | <https://wwwnc.cdc.gov/travel/yellowbook/2024/infections-diseases/smallpox-other-orthopoxvirus-associated-infections> | Details on smallpox and related infections, including vaccination information. | Important for understanding risks associated with orthopoxviruses. |
| 66 | CDC - Tick-borne Encephalitis | <https://wwwnc.cdc.gov/travel/yellowbook/2024/infections-diseases/tick-borne-encephalitis> | Guidelines on tick-borne encephalitis, prevalent in certain regions. | Vital for travelers to tick-endemic areas in Europe and Asia. |
| 67 | CDC - Varicella (Chickenpox) | <https://wwwnc.cdc.gov/travel/yellowbook/2024/infections-diseases/varicella-chickenpox> | Comprehensive guide on chickenpox, including vaccine recommendations. | Essential for ensuring immunity, especially for non-immune travelers. |
| 68 | CDC - Viral Hemorrhagic Fevers | <https://wwwnc.cdc.gov/travel/yellowbook/2024/infections-diseases/viral-hemorrhagic-fevers> | Information on various viral hemorrhagic fevers, focusing on prevention and risk. | Critical for travel to areas with outbreaks of diseases like Ebola and Marburg. |
| 69 | CDC - Yellow Fever | <https://wwwnc.cdc.gov/travel/yellowbook/2024/infections-diseases/yellow-fever> | Details on yellow fever, including vaccine advice and areas of risk. | Crucial for travel to yellow fever-endemic regions in Africa and South America. |
| 70 | CDC - Zika Virus | <https://wwwnc.cdc.gov/travel/yellowbook/2024/infections-diseases/zika> |  |  |
| 71 | CDC - Amebiasis | <https://wwwnc.cdc.gov/travel/yellowbook/2024/infections-diseases/amebiasis> | Information on amebiasis, including symptoms, transmission, and prevention. | Important for travelers to areas with poor sanitation and hygiene. |
| 72 | CDC - Angiostrongyliasis | <https://wwwnc.cdc.gov/travel/yellowbook/2024/infections-diseases/angiostrongyliasis> | Overview of angiostrongyliasis, a parasitic infection, focusing on prevention for travelers. | Relevant for travel to endemic regions, particularly in Southeast Asia and the Pacific Islands. |
| 73 | CDC - Cryptosporidiosis | <https://wwwnc.cdc.gov/travel/yellowbook/2024/infections-diseases/cryptosporidiosis> | Guidelines on cryptosporidiosis, a parasitic disease, including prevention tips. | Crucial for managing risks associated with waterborne illnesses. |
| 74 | CDC - Cutaneous Larva Migrans | <https://wwwnc.cdc.gov/travel/yellowbook/2024/infections-diseases/cutaneous-larva-migrans> | Information on cutaneous larva migrans, a skin infection caused by hookworms. | Important for travelers to tropical and subtropical beaches. |
| 75 | CDC - Cyclosporiasis | <https://wwwnc.cdc.gov/travel/yellowbook/2024/infections-diseases/cyclosporiasis> | Advice on cyclosporiasis, focusing on prevention and treatment. | Relevant for travelers to regions where the parasite is endemic, particularly during rainy seasons. |
| 76 | CDC - Cysticercosis | <https://wwwnc.cdc.gov/travel/yellowbook/2024/infections-diseases/cysticercosis> | Comprehensive guide on cysticercosis, including transmission, symptoms, and prevention. | Essential for understanding the risks and prevention of this parasitic infection, especially in developing countries. |
| 77 | CDC - Echinococcosis | <https://wwwnc.cdc.gov/travel/yellowbook/2024/infections-diseases/echinococcosis> | Information about echinococcosis, a parasitic disease, including prevention for travelers. | Important for understanding risks associated with Echinococcus tapeworms, especially in rural areas. |
| 78 | CDC - Enterobiasis (Pinworm) | <https://wwwnc.cdc.gov/travel/yellowbook/2024/infections-diseases/enterobiasis-pinworm> | Guidelines on pinworm infection, focusing on prevention and treatment. | Relevant for travelers, especially those in close-contact settings like hostels or camps. |
| 79 | CDC - Filariasis, Lymphatic | <https://wwwnc.cdc.gov/travel/yellowbook/2024/infections-diseases/filariasis-lymphatic> | Overview of lymphatic filariasis, including transmission and prevention tips. | Crucial for travel to tropical regions where the disease is endemic. |
| 80 | CDC - Flukes, Liver | <https://wwwnc.cdc.gov/travel/yellowbook/2024/infections-diseases/flukes-liver> | Information on liver flukes, including risks and preventive measures. | Important for travelers to East Asia and other areas where liver flukes are common. |
| 81 | CDC - Flukes, Lung | <https://wwwnc.cdc.gov/travel/yellowbook/2024/infections-diseases/flukes-lung> | Guidelines on lung flukes, focusing on transmission and prevention for travelers. | Relevant for travel to Southeast Asia and parts of Africa where lung flukes are found. |
| 82 | CDC - Giardiasis | <https://wwwnc.cdc.gov/travel/yellowbook/2024/infections-diseases/giardiasis> | Comprehensive advice on giardiasis, a common cause of traveler's diarrhea. | Essential for travelers to understand prevention and treatment of giardiasis. |
| 83 | CDC - Helminths, Soil-Transmitted | <https://wwwnc.cdc.gov/travel/yellowbook/2024/infections-diseases/helminths-soil-transmitted> | Information on soil-transmitted helminths, including prevention strategies. | Important for travelers to areas with poor sanitation, where these parasites are prevalent. |
| 84 | CDC - Leishmaniasis, Cutaneous | <https://wwwnc.cdc.gov/travel/yellowbook/2024/infections-diseases/leishmaniasis-cutaneous> | Overview of cutaneous leishmaniasis, focusing on transmission and prevention. | Relevant for travel to tropical and subtropical regions where the disease is endemic. |
| 85 | CDC - Leishmaniasis, Visceral | <https://wwwnc.cdc.gov/travel/yellowbook/2024/infections-diseases/leishmaniasis-visceral> | Guidelines on visceral leishmaniasis, including risks and preventive measures. | Crucial for understanding risks associated with the most severe form of leishmaniasis. |
| 86 | CDC - Malaria | <https://wwwnc.cdc.gov/travel/yellowbook/2024/infections-diseases/malaria> | Comprehensive advice on malaria, including prevention, medication, and areas of risk. | Essential for travelers to malaria-endemic regions. |
| 87 | CDC - Onchocerciasis (River Blindness) | <https://wwwnc.cdc.gov/travel/yellowbook/2024/infections-diseases/onchocerciasis-river-blindness> | Information on river blindness, focusing on transmission and prevention for travelers. | Important for travel to Sub-Saharan Africa and parts of Central and South America. |
| 88 | CDC - Sarcocystosis | <https://wwwnc.cdc.gov/travel/yellowbook/2024/infections-diseases/sarcocystosis> | Overview of sarcocystosis, including transmission risks and symptoms. | Relevant for understanding this less common parasitic infection. |
| 89 | CDC - Scabies | <https://wwwnc.cdc.gov/travel/yellowbook/2024/infections-diseases/scabies> | Guidelines on scabies prevention and treatment, particularly for travelers. | Useful for managing scabies, a common skin infestation in crowded living conditions. |
| 90 | CDC - Schistosomiasis | <https://wwwnc.cdc.gov/travel/yellowbook/2024/infections-diseases/schistosomiasis> | Comprehensive information on schistosomiasis, including prevention and areas of risk. | Vital for travelers to freshwater bodies in tropical and subtropical regions. |
| 91 | CDC - Strongyloidiasis | <https://wwwnc.cdc.gov/travel/yellowbook/2024/infections-diseases/strongyloidiasis> | Overview of strongyloidiasis, a parasitic infection, with prevention tips. | Important for understanding risks in regions with poor sanitation. |
| 92 | CDC - Taeniasis | <https://wwwnc.cdc.gov/travel/yellowbook/2024/infections-diseases/taeniasis> | Information on taeniasis, caused by tapeworms, including transmission and prevention. | Relevant for travelers to areas where tapeworm infection is prevalent. |
| 93 | CDC - Toxoplasmosis | <https://wwwnc.cdc.gov/travel/yellowbook/2024/infections-diseases/toxoplasmosis> | Guidelines on toxoplasmosis, focusing on prevention and risk factors for travelers. | Crucial for understanding risks associated with this parasitic infection. |
| 94 | CDC - Trypanosomiasis, African | <https://wwwnc.cdc.gov/travel/yellowbook/2024/infections-diseases/trypanosomiasis-african> | Details on African trypanosomiasis (sleeping sickness), including prevention. | Important for travel to Sub-Saharan Africa where the disease is endemic. |
| 95 | CDC - Trypanosomiasis, American (Chagas Disease) | <https://wwwnc.cdc.gov/travel/yellowbook/2024/infections-diseases/trypanosomiasis-american-chagas-disease> | Comprehensive information on Chagas disease, including transmission, prevention, and areas of risk. | Vital for travelers to Latin America where the disease is transmitted by insects. |
| 96 | CDC - Coccidioidomycosis (Valley Fever) | <https://wwwnc.cdc.gov/travel/yellowbook/2024/infections-diseases/coccidioidomycosis-valley-fever> | Information on Valley Fever, focusing on risks in endemic areas and prevention. | Important for travelers to southwestern United States and parts of Central and South America. |
| 97 | CDC - Histoplasmosis | <https://wwwnc.cdc.gov/travel/yellowbook/2024/infections-diseases/histoplasmosis> | Guidelines on histoplasmosis, a fungal infection, including prevention and risk areas. | Relevant for travelers to areas with bat and bird droppings, particularly in cave environments. |
| 98 | CDC - Pregnant Travelers | <https://wwwnc.cdc.gov/travel/yellowbook/2024/family/pregnant-travelers> | Comprehensive advice for pregnant travelers, covering health risks and precautions. | Crucial for managing health and safety of pregnant travelers. |
| 99 | CDC - Breastfeeding | <https://wwwnc.cdc.gov/travel/yellowbook/2024/family/breastfeeding> | Information on breastfeeding while traveling, including tips and health considerations. | Important for breastfeeding mothers to ensure safe and healthy travel. |
| 100 | CDC - Infants and Children | <https://wwwnc.cdc.gov/travel/yellowbook/2024/family/infants-and-children> | Guidelines for traveling with infants and children, focusing on health and safety. | Essential for families traveling with young children, addressing specific needs and risks. |
| 101 | CDC - Vaccine Recommendations for Infants and Children | <https://wwwnc.cdc.gov/travel/yellowbook/2024/family/vaccine-recommendations-for-infants-and-children> | Vaccine recommendations for infants and children when traveling. | Vital for ensuring proper vaccination of young travelers. |
| 102 | CDC - Pets and Service Animals | <https://wwwnc.cdc.gov/travel/yellowbook/2024/family/pets-and-service-animals> | Information on traveling with pets and service animals, including health and documentation requirements. | Useful for travelers who plan to bring pets or service animals on their trips. |
| 103 | CDC - Air Travel | <https://wwwnc.cdc.gov/travel/yellowbook/2024/air-land-sea/air-travel> | Comprehensive guidelines on health considerations for air travel. | Important for understanding health risks and precautions associated with flying. |
| 104 | CDC - Deep Vein Thrombosis and Pulmonary Embolism | <https://wwwnc.cdc.gov/travel/yellowbook/2024/air-land-sea/deep-vein-thrombosis-and-pulmonary-embolism> | Information on the risks of deep vein thrombosis (DVT) and pulmonary embolism during air travel. | Crucial for travelers on long flights to manage risks of blood clots. |
| 105 | CDC - Jet Lag | <https://wwwnc.cdc.gov/travel/yellowbook/2024/air-land-sea/jet-lag> | Advice on managing jet lag, including prevention and treatment strategies. | Useful for travelers crossing multiple time zones. |
| 106 | CDC - Road and Traffic Safety | <https://wwwnc.cdc.gov/travel/yellowbook/2024/air-land-sea/road-and-traffic-safety> | Guidelines on road and traffic safety for travelers, including driving in foreign countries. | Vital for travelers using road transportation, emphasizing safety and local laws. |
| 107 | CDC - Cruise Ship Travel | <https://wwwnc.cdc.gov/travel/yellowbook/2024/air-land-sea/cruise-ship-travel> | Health considerations for cruise ship travel, including common health issues and prevention. | Important for cruise passengers, covering a range of health topics specific to cruise travel. |
| 108 | CDC - Motion Sickness | <https://wwwnc.cdc.gov/travel/yellowbook/2024/air-land-sea/motion-sickness> | Information on preventing and managing motion sickness during travel. | Useful for travelers prone to motion sickness on various modes of transportation. |
| 109 | CDC - Visiting Friends and Relatives | <https://wwwnc.cdc.gov/travel/yellowbook/2024/work-and-other-reasons/visiting-friends-and-relatives> | Health advice for travelers visiting friends and relatives, often in their country of origin. | Important for understanding specific health risks and preventive measures in these scenarios. |
| 110 | CDC - Mass Gatherings | <https://wwwnc.cdc.gov/travel/yellowbook/2024/work-and-other-reasons/mass-gatherings> | Guidelines on health considerations for attending mass gatherings. | Crucial for managing health risks at events with large crowds, like festivals or sporting events. |
| 111 | CDC - Sex and Travel | <https://wwwnc.cdc.gov/travel/yellowbook/2024/work-and-other-reasons/sex-and-travel> | Information on safe sex practices and risks related to sexual activity during travel. | Vital for promoting safe and responsible sexual behavior while traveling. |
| 112 | CDC - African Safaris | <https://wwwnc.cdc.gov/travel/yellowbook/2024/itineraries/african-safaris> | Health tips and precautions for travelers on African safaris. | Useful for those planning safari adventures, covering risks like malaria and animal encounters. |
| 113 | CDC - Egypt | <https://wwwnc.cdc.gov/travel/yellowbook/2024/itineraries/egypt> | Travel health information specific to visitors to Egypt. | Important for understanding the unique health considerations and risks when traveling to Egypt. |
| 114 | CDC - Saudi Arabia: Hajj and Umrah Pilgrimages | <https://wwwnc.cdc.gov/travel/yellowbook/2024/itineraries/saudi-arabia-hajj-and-umrah-pilgrimages> | Guidelines for those traveling to Saudi Arabia for Hajj or Umrah. | Essential for pilgrims, covering health risks and preventive measures specific to these religious gatherings. |
| 115 | CDC - South Africa | <https://wwwnc.cdc.gov/travel/yellowbook/2024/itineraries/south-africa> | Travel health advice for visitors to South Africa. | Useful for travelers to South Africa, with information on various health risks and precautions. |
| 116 | CDC - Tanzania and Zanzibar | <https://wwwnc.cdc.gov/travel/yellowbook/2024/itineraries/tanzania-and-zanzibar> | Health considerations for travelers to Tanzania and Zanzibar. | Important for travelers to these destinations, covering risks like malaria and traveler's diarrhea. |
| 117 | CDC - Brazil | <https://wwwnc.cdc.gov/travel/yellowbook/2024/itineraries/brazil> | Travel health information specific to visitors to Brazil. | Important for understanding health risks and precautions in Brazil. |
| 118 | CDC - Cusco and Machu Picchu | <https://wwwnc.cdc.gov/travel/yellowbook/2024/itineraries/cusco-and-machu-picchu> | Health advice for travelers visiting Cusco and Machu Picchu in Peru. | Useful for managing high altitude health risks and other local considerations. |
| 119 | CDC - Dominican Republic | <https://wwwnc.cdc.gov/travel/yellowbook/2024/itineraries/dominican-republic> | Health considerations for travelers to the Dominican Republic. | Covers risks like mosquito-borne diseases and traveler's diarrhea. |
| 120 | CDC - Guatemala and Belize | <https://wwwnc.cdc.gov/travel/yellowbook/2024/itineraries/guatemala-and-belize> | Travel health guidelines for visitors to Guatemala and Belize. | Essential for understanding regional health concerns, including food and water safety. |
| 121 | CDC - Haiti | <https://wwwnc.cdc.gov/travel/yellowbook/2024/itineraries/haiti> | Health information for travelers to Haiti, focusing on a range of health risks. | Important for visitors to Haiti, addressing specific local health challenges. |
| 122 | CDC - Iguacu Falls | <https://wwwnc.cdc.gov/travel/yellowbook/2024/itineraries/iguacu-falls> | Tips and health advice for travelers visiting Iguacu Falls. | Useful for travelers to this popular destination, covering environmental and health considerations. |
| 123 | CDC - Jamaica | <https://wwwnc.cdc.gov/travel/yellowbook/2024/itineraries/jamaica> | Travel health guidelines for Jamaica, including vaccinations and disease prevention. | Vital for travelers to Jamaica, with advice on common health issues in the region. |
| 124 | CDC - Mexico | <https://wwwnc.cdc.gov/travel/yellowbook/2024/itineraries/mexico> | Comprehensive health information for travelers to Mexico. | Covers a wide range of health considerations, from food and water safety to mosquito-borne diseases. |
| 125 | CDC - Peru | <https://wwwnc.cdc.gov/travel/yellowbook/2024/itineraries/peru> | Health advice for visitors to Peru, including high-altitude destinations. | Important for managing health risks associated with travel in various regions of Peru. |
| 126 | CDC - Burma (Myanmar) | <https://wwwnc.cdc.gov/travel/yellowbook/2024/itineraries/burma-myanmar> | Health advice for travelers to Burma (Myanmar), including prevalent health risks. | Important for understanding specific health considerations in Burma (Myanmar). |
| 127 | CDC - Cambodia | <https://wwwnc.cdc.gov/travel/yellowbook/2024/itineraries/cambodia> | Travel health information for visitors to Cambodia. | Covers health risks and preventive measures for travel in Cambodia. |
| 128 | CDC - China | <https://wwwnc.cdc.gov/travel/yellowbook/2024/itineraries/china> | Comprehensive guidelines for travelers visiting China. | Vital for addressing a wide range of health concerns in various regions of China. |
| 129 | CDC - India | <https://wwwnc.cdc.gov/travel/yellowbook/2024/itineraries/india> | Health considerations for travelers to India, including vaccinations and disease prevention. | Essential for managing health risks in the diverse travel environments of India. |
| 130 | CDC - Nepal | <https://wwwnc.cdc.gov/travel/yellowbook/2024/itineraries/nepal> | Guidelines for travelers visiting Nepal, with a focus on high-altitude health risks. | Useful for trekkers and travelers to Nepal, particularly those visiting the Himalayas. |
| 131 | CDC - Thailand | <https://wwwnc.cdc.gov/travel/yellowbook/2024/itineraries/thailand> | Travel health advice for visitors to Thailand, including urban and rural considerations. | Important for understanding health risks and preventive measures in Thailand. |
| 132 | CDC - Vietnam | <https://wwwnc.cdc.gov/travel/yellowbook/2024/itineraries/vietnam> | Health information for travelers to Vietnam, covering common risks and precautions. | Relevant for visitors to Vietnam, addressing regional health issues and safety. |
| 133 | CDC - General Approach to the Returned Traveler | <https://wwwnc.cdc.gov/travel/yellowbook/2024/posttravel-evaluation/general-approach-to-the-returned-traveler> | Guidelines on evaluating health issues in travelers after returning from international travel. | Essential for understanding common post-travel health issues and appropriate responses. |
| 134 | CDC - Rapid Diagnostic Tests for Infectious Diseases | <https://wwwnc.cdc.gov/travel/yellowbook/2024/posttravel-evaluation/rapid-diagnostic-tests-for-infectious-diseases> | Information on the use of rapid diagnostic tests for identifying infectious diseases in returned travelers. | Useful for timely diagnosis and treatment of travel-related infectious diseases. |
| 135 | CDC - Fever in the Returned Traveler | <https://wwwnc.cdc.gov/travel/yellowbook/2024/posttravel-evaluation/fever-in-the-returned-traveler> | Advice on the assessment and management of fever in travelers after returning from abroad. | Important for identifying potential causes of fever and related health concerns post-travel. |
| 136 | CDC - Respiratory Infections in Returned Travelers | <https://wwwnc.cdc.gov/travel/yellowbook/2024/posttravel-evaluation/respiratory-infections> | Guidelines on evaluating and managing respiratory infections in travelers returning from their trips. | Relevant for addressing common respiratory issues encountered after travel. |
| 137 | CDC - Persistent Diarrhea in Returned Travelers | <https://wwwnc.cdc.gov/travel/yellowbook/2024/posttravel-evaluation/persistent-diarrhea-in-returned-travelers> | Information on causes and treatment of persistent diarrhea in travelers after their return. | Crucial for managing extended gastrointestinal issues post-travel. |
| 138 | CDC - Dermatologic Conditions in Returned Travelers | <https://wwwnc.cdc.gov/travel/yellowbook/2024/posttravel-evaluation/dermatologic-conditions> | Overview of common skin conditions in travelers returning from trips, including treatment options. | Useful for diagnosing and treating skin problems that may arise after travel. |
| 139 | CDC - Sexually Transmitted Infections in Returned Travelers | <https://wwwnc.cdc.gov/travel/yellowbook/2024/posttravel-evaluation/sexually-transmitted-infections> | Guidelines for the evaluation and management of sexually transmitted infections in returned travelers. | Important for addressing sexual health concerns post-international travel. |
| 140 | CDC - Newly Arrived Immigrants, Refugees, and Other Migrants | <https://wwwnc.cdc.gov/travel/yellowbook/2024/posttravel-evaluation/newly-arrived-immigrants-refugees-and-other-migrants> | Health considerations and guidelines for newly arrived immigrants, refugees, and other migrants. | Relevant for understanding specific health issues and needs of this group post-arrival. |
| 141 | Italian Ministry of Health - Healthcare for EU Travelers | <https://www.salute.gov.it/portale/cureUE/dettaglioContenutiCureUE.jsp?lingua=italiano&id=897&area=cureUnioneEuropea&menu=vuoto> | Information on healthcare options and rights for travelers within the EU. | Essential for Italian and EU citizens traveling within the European Union, providing details on healthcare access. |
| 142 | Viaggiare Sicuri | <https://www.viaggiaresicuri.it/home> | Official Italian portal offering travel safety advice and updates. | Offers comprehensive travel advisories, safety tips, and country-specific information for Italian travelers. |
| 143 | Dove Siamo Nel Mondo | <https://www.dovesiamonelmondo.it/home.html> | A service to assist Italian citizens traveling abroad. | Allows travelers to notify their whereabouts to the Italian Ministry of Foreign Affairs for safety and assistance. |
| 144 | Italian Ministry of Health - Infectious Diseases for Travelers | <https://www.salute.gov.it/portale/malattieInfettive/dettaglioContenutiMalattieInfettive.jsp?lingua=italiano&id=653&area=Malattie%20infettive&menu=viaggiatori> | Information on infectious diseases and precautions for travelers. | Provides guidance on preventive measures and risks of infectious diseases for Italian travelers. |
|  |  |  |  |  |
