# Supplementary material for: A decalogue for personalized travel health assistance with AI-driven chatbots
Source: J Travel Med. 2024 Feb 12;31(4):taae026. doi: 10.1093/jtm/taae026 (PMC11149716; doi:10.1093/jtm/taae026)
Supplement: S3_JTM_Baglivo_taae026 [file s3_jtm_baglivo_taae026.docx]

# List of Italian travel medicine clinics

This document is a vital resource for the Italian Travel Medicine Advisor, particularly when responding to inquiries related to:

1. **Locations for Vaccinations**: If a user requests information about where to get vaccinations in Italy, especially for pre-travel health preparations.

2. **Travel Health Information and Consultation**: When a user seeks advice on travel health or needs to consult about pre-travel health concerns.

3. **Authorized Centers for Yellow Fever Vaccinations**: This document lists centers authorized under the Italian Ministry of Health decree dated August 2, 2023 (Decree 23A05095), which certifies centers for administering yellow fever vaccinations and issuing the corresponding certificates.

Use this document as a reference to guide users to the nearest and most suitable travel medicine centers in Italy. It is an authoritative source for up-to-date information on the location and services of these centers, ensuring that travelers receive the necessary health care and vaccinations before their journeys.

| **Regione** | **DENOMINAZIONE CENTRO** | **Città** | **Indirizzo** | **Telefono** | **E-mail** |
| --- | --- | --- | --- | --- | --- |
| **ABRUZZO** | Azienda USL 1 Avezzano-Sulmona– L’Aquila | Avezzano | Via Monte Velino 14 | tel 0863 499815 |  |
| **ABRUZZO** | Ambulatorio vaccinale  c/o P.O. SS Annunziata | Chieti | Via Padre Andrea  Valignani | tel 0871 345196 |  |
| **ABRUZZO** | Azienda USL 1 Avezzano-Sulmona–  L’Aquila c/o Ospedale Regionale S. Salvatore | L'Aquila | Via Lorenzo Natali 1 – Loc. Coppito | tel 0862 368834 |  |
| **ABRUZZO** | Azienda USL Chieti 2 | Ortona | P.zza San Francesco 2 | tel 085 9173226 |  |
| **ABRUZZO** | USMAF SASN di Lazio, Marche, Umbria, Abruzzo e Molise - Unità  Territoriale di Pescara | Pescara | Lungomare C. Colombo 4 | tel 065994 4741 | [usma.pescara@sanita.it](mailto:usma.pescara@sanita.it) |
| **ABRUZZO** | Azienda USL di Pescara | Pescara | Via Paolini 45 – Palazzina G, Ingresso 3 | tel 085 4253959 |  |
| **ABRUZZO** | Azienda USL 1 Avezzano-Sulmona– L’Aquila | Sulmona | Via Gorizia 4 | tel 0864 499601 |  |
| **ABRUZZO** | Azienda Asl - SIESP Centro di Medicina dei Viaggi | Teramo | C.da Casalena Teramo | tel 0861 420569 |  |
| **ABRUZZO** | Azienda USL 3 Lanciano | Vasto | Via Michetti 86 | tel 0873 308732 |  |
| **BASILICATA** | ASL Centro Vaccinale | Lauria | Via XXV Aprile - c/o Presidio Ospedaliero di Lauria | tel 0973 621438 -  621443 |  |
| **BASILICATA** | ASL Centro Vaccinale | Matera | Via Montescaglioso | tel 0835 253609 -  569 |  |
| **BASILICATA** | ASL Centro Vaccinale | Potenza | Via della Fisica 18 A/B | tel 0971 425227 -  31 |  |
| **BASILICATA** | ASL Centro Vaccinale | Rionero in Vulture | Via Madre Miradio della Provvidenza | tel 0972 773948 -  50 |  |
| **CALABRIA** | Centro di medicina del Viaggiatore – Servizio  Vaccinazioni Catanzaro | Catanzaro | Viale Pio X ex clinica Mater Dei | tel 0961 703481 -  741948 |  |
| **CALABRIA** | Centro per la Medicina dei Viaggi e delle Migrazioni – ASP Cosenza | Cosenza | Via Alimena | tel 0984 8931 –  0984 893587 |  |
| **CALABRIA** | Travel Medicine Cetraro | Cosenza | Località Testa | tel 0982 977417 |  |
| **CALABRIA** | U.O. Igiene e Sanità Pubblica | Lamezia Terme | Via Sottotenente Notaro | Tel 0968 208304  - 41 |  |
| **CALABRIA** | USMAF SASN di  Puglia, Calabria e Basilicata - Unità Territoriale di RC | Reggio Calabria | Svincolo Autostradale Porto Piazzale Nord | tel 06 5994 4830 | [usma.reggiocalabria@sanita.it](mailto:usma.reggiocalabria@sanita.it) |
| **CALABRIA** | Centro di Medicina del Viaggiatore c/o ufficio medicina preventiva ex  A.S. Locri | Reggio Calabria | Via De Gasperi - Ardore | tel 0964 628179 |  |
| **CALABRIA** | Medicina del Viaggiatore | Reggio Calabria | Via Calabria traversa III n. 8 R.C. | Tel 0965 347353  - 56 |  |
| **CALABRIA** | Centro di medicina dei Viaggi - ASP Vibo Valentia c/o struttura di  medicina preventiva | Vibo Valentia | Via Moderata Durant | tel 0963 962561 |  |
| **CAMPANIA** | Asl 2 Centro di Medicina del Viaggiatore e  Vaccinazioni Internazionali | Avellino | Via Circumvallazione 77 | tel 0825 292665 |  |
| **CAMPANIA** | Asl Benevento | Benevento | Via Mascellaro 1 | tel 0824 308367 |  |
| **CAMPANIA** | USMAF SASN  Campania e Sardegna – | Capodichino | Viale F.R. di Calabria – Pal. Pegaso - II piano - | tel 06 5994 4707 | [usma.napoli.capodichino@sani](mailto:usma.napoli.capodichino@sanita.it) [ta.it](mailto:usma.napoli.capodichino@sanita.it) |

|  | Unità Territoriale di Capodichino |  | Aeroporto internazionale di Napoli |  |  |
| --- | --- | --- | --- | --- | --- |
| **CAMPANIA** | Asl Napoli 3 sud | Castellamare di Stabia | Corso Alcide De Gasperi 167 | tel 081 8729822-  824 - 271 |  |
| **CAMPANIA** | Asl Caserta SEP Centro di Medicina del Viaggiatore | Marcianise | Piazza Carità | tel 0823 350927 |  |
| **CAMPANIA** | USMAF SASN  Campania e Sardegna –  Unità Territoriale di Napoli | Napoli | Via Immacolatella Vecchia - Interno porto snc | tel 06 5994 4703 | [usma.napoliporto.ambulatorio](mailto:usma.napoliporto.ambulatorio@sanita.it) [@sanita.it](mailto:usma.napoliporto.ambulatorio@sanita.it) |
| **CAMPANIA** | Asl Napoli 1 centro | Napoli | Piazza Municipio, 84  3° piano | tel. 081 2547088-  7086 | [uosd.prevcoll24-](mailto:uosd.prevcoll24-31@aslnapoli1centro.it) [31@aslnapoli1centro.it](mailto:uosd.prevcoll24-31@aslnapoli1centro.it) |
| **CAMPANIA** | Asl Napoli 2 nord – ADEP-UOSE n 1 | Napoli | Corso Campano 316- Giugliano | tel 081 18843024 | [uose3@aslnapoli2nord.it](mailto:uose3@aslnapoli2nord.it) |
| **CAMPANIA** | USMAF SASN  Campania e Sardegna – Unità Territoriale di Salerno | Salerno | Molo Manfredi snc - Interno Porto Salerno | tel 06 5994-  4835-9695 | [usma.salernoporto.ambulatorio](mailto:usma.salernoporto.ambulatorio@sanita.it) [@sanita.it](mailto:usma.salernoporto.ambulatorio@sanita.it) ; [usma.salerno@sanita.it](mailto:usma.salerno@sanita.it) |
| **CAMPANIA** | Asl Salerno c/o distretto 64 | Salerno | Via Vernieri 14 | tel 089 693647 |  |
| **EMILIA ROMAGNA** | ASL BOLOGNA | Osp. SS Salvatore | Via Enzo Palma 1 | tel 800 884888  (CUP) - 051  6813303 |  |
| **EMILIA ROMAGNA** | ASL RAVENNA | Bagnacavallo | Via Vittorio Veneto 8 | tel 0545 283055 |  |
| **EMILIA ROMAGNA** | ASL BOLOGNA | Bentivoglio | Via Marconi, 35 | tel 051 6644711  – 6644641 |  |
| **EMILIA ROMAGNA** | ASL BOLOGNA | Bologna | Via Gramsci 12 | tel 051 6079745 -  6079740 |  |
| **EMILIA ROMAGNA** | ASL BOLOGNA Loc.  Bazzano, Ospedale Dossetti | Bologna | Viale dei martiri 10 B | 051 596970  segreteria Tel 800 88488  prenotazioni |  |
| **EMILIA ROMAGNA** | USMAF SASN di  Toscana, Emilia Romagna – Ufficio Territoriale di Bologna | Bologna | Aeroporto G. Marconi Via del Triumvirato 84 -  B.go Panigale - | tel 06 5994 4709 | [usma.bologna@sanita.it](mailto:usma.bologna@sanita.it) |
| **EMILIA ROMAGNA** | ASL PIACENZA | Borgonovo Val Tidone | Via Seminò 20 | tel 0523 880520 -  800651941 |  |
| **EMILIA ROMAGNA** | ASL PARMA | Borgotaro | Via Benefattori 12 - Ospedale Borgotaro, | tel 0525 970328  – 32, 0525  300424 |  |
| **EMILIA ROMAGNA** | ASL BOLOGNA | Budrio | Via Benni 44 | tel 800 884888  (CUP) - 051  809882 |  |
| **EMILIA ROMAGNA** | ASL MODENA | Carpi | Piazzale Donatori del Sangue 3 | tel 059 659912 |  |
| **EMILIA ROMAGNA** | ASL BOLOGNA | Casalecchio di Reno | Piazzale Rita Levi Montalcini 5 | tel 800 884888 (CUP) |  |
| **EMILIA ROMAGNA** | ASL MODENA | Castel Franco Emilia | Piazzale Grazia Deledda | tel 059 929159 |  |
| **EMILIA ROMAGNA** | ASL REGGIO EMILIA | Castelnovo ne’ Monti | Via Roma 26 | tel 0522 617343 |  |
| **EMILIA ROMAGNA** | ASL RIMINI | Cattolica | P.zza della Repubblica 18 | tel 0541 834242 |  |
| **EMILIA ROMAGNA** | ASL RAVENNA | Cervia | Via dell’Ospedale 17 | tel 0544 287641 |  |
| **EMILIA ROMAGNA** | ASL CESENA | Cesena | P.zza Anna Magnani 146 | tel 0547 352416 |  |
| **EMILIA**  **ROMAGNA** | ASL REGGIO EMILIA | Correggio | Piazza S. Rocco 4 | tel 0522 630451 |  |
| **EMILIA ROMAGNA** | ASL RAVENNA | Faenza | Via Zaccagnini 22 | tel 0546 602520 -  21 |  |
| **EMILIA ROMAGNA** | ASL FERRARA | Ferrara | Via G.Bianchi 4 (Ex Motovelodromo) | 800 532000 | [vaccinazioniadulti@ausl.fe.it](mailto:vaccinazioniadulti@ausl.fe.it) |
| **EMILIA**  **ROMAGNA** | ASL FERRARA  Ospedale SS Annunziata | Ferrara | Via Vicini 2 | tel 800 532000 |  |

| **EMILIA ROMAGNA** | ASL FERRARA Casa  della Salute | Ferrara | Via Felletti 2 | tel 800 532000 |  |
| --- | --- | --- | --- | --- | --- |
| **EMILIA**  **ROMAGNA** | ASL PARMA | Fidenza | Via Don Tincati 5/M | tel 0524 515515 |  |
| **EMILIA ROMAGNA** | ASL PIACENZA | Fiorenzuola D’Arda | Via Roma 12 | tel 800651941 |  |
| **EMILIA ROMAGNA** | ASL FORLI’ | Forlì | Via della Rocca 19 | tel 0543 733527 |  |
| **EMILIA**  **ROMAGNA** | ASL REGGIO EMILIA | Guastalla | Piazza Matteotti 4 | tel 0522 837612 |  |
| **EMILIA ROMAGNA** | ASL IMOLA Azienda USL | Imola | Via Amendola 8 | tel 0542 604916 |  |
| **EMILIA ROMAGNA** | ASL PARMA | Langhirano | Via Roma 42/1 | tel 0521 865302 -  0521 865304 |  |
| **EMILIA ROMAGNA** | ASL RAVENNA | Lugo | Viale Masi 20 | tel 0545 283055 |  |
| **EMILIA ROMAGNA** | ASL MODENA | Mirandola | Via L. Smerieri 3 | tel 0535 602873 |  |
| **EMILIA ROMAGNA** | ASL MODENA | Modena | St. Da Martiniana 21 | tel 059 3963155 |  |
| **EMILIA ROMAGNA** | ASL REGGIO EMILIA | Montecchio E. | Via Marconi 18 | tel 0522 860175 |  |
| **EMILIA ROMAGNA** | ASL PARMA | Noceto | Via Gen. C.A. Dalla Chiesa 5/A | tel 0521 667412 |  |
| **EMILIA ROMAGNA** | ASL PARMA | Parma | Via Vasari 13/A | tel 0521 396437 |  |
| **EMILIA ROMAGNA** | ASL MODENA | Pavullo nel Frignano | Viale dei Martiri 63 | tel 0536 29359 |  |
| **EMILIA ROMAGNA** | ASL PIACENZA | Piacenza | Piazzale Milano 2 | tel 0523 317826 |  |
| **EMILIA ROMAGNA** | ASL BOLOGNA | Pianoro | Viale Risorgimento 8 | tel 800 884888  (CUP) - 051  776050 |  |
| **EMILIA**  **ROMAGNA** | ASL BOLOGNA | Porretta Terme | Via Oreste Zagnoni,  1 | tel 0534 208221 |  |
| **EMILIA ROMAGNA** | ASL RAVENNA | Ravenna | Via Fiume Abbandonato 134 | tel 0544 286686 |  |
| **EMILIA ROMAGNA** | USMAF SASN di  Toscana, Emilia Romagna – Ufficio Territoriale di Ravenna | Ravenna | Via Rondinelli 6 | tel 06 5994 4712 | [usma.ravenna@sanita.it](mailto:usma.ravenna@sanita.it) |
| **EMILIA ROMAGNA** | ASL REGGIO EMILIA | Reggio Emilia | Via Amendola 2 | tel 0522 335749 -  700 |  |
| **EMILIA ROMAGNA** | ASL RIMINI | Riccione | Via San Miniato 16 | tel 0541 668386 |  |
| **EMILIA ROMAGNA** | ASL RIMINI | Rimini | Via Coriano 38 | tel 0541 707213 -  707290 |  |
| **EMILIA ROMAGNA** | ASL BOLOGNA | S. Lazzaro di Savena | Via Repubblica 11 | tel 051 6224294 |  |
| **EMILIA ROMAGNA** | ASL PARMA | San Secondo Parmense | P.zza Martiri della libertà 24 | tel 0521 371743 |  |
| **EMILIA ROMAGNA** | ASL RIMINI | Santarcangelo di Romagna | P.zza Suor Angela Molari | tel 0541 326557 |  |
| **EMILIA ROMAGNA** | ASL MODENA | Sassuolo | Via F.lli Cairoli, 19 | tel 0536 863725 |  |
| **EMILIA ROMAGNA** | ASL REGGIO EMILIA | Scandiano | Via Martiri della Libertà 8 | tel 0522 850304 |  |
| **EMILIA ROMAGNA** | ASL MODENA | Vignola | Via Libertà 799 | tel 059 777044 |  |
| **EMILIA ROMAGNA** | ASL BOLOGNA | Zola Predosa | Piazza di Vittorio 1 | tel 800 884888  (CUP) – 051  6188924 |  |
| **FRIULI-VENEZIA GIULIA** | ASUFC - Codroipo | Codroipo | Viale Duodo 82 | tel. 0432 909180 | [segr.dip@asufc.sanita.fvg.it](mailto:segr.dip@asufc.sanita.fvg.it) |
| **FRIULI-VENEZIA GIULIA** | ASUFC - Gemona del Friuli | Gemona del Friuli | Piazza Rodolone 1 | tel 0432 989338 | [segr.dip@asufc.sanita.fvg.it](mailto:segr.dip@asufc.sanita.fvg.it) |
| **FRIULI-VENEZIA GIULIA** | ASUGI – Centro Vaccinale | Gorizia | Via Vittorio Veneto 173 | tel 0481 592818 | [vaccinazionigo@asugi.sanita.f](mailto:vaccinazionigo@asugi.sanita.fvg.it) [vg.it](mailto:vaccinazionigo@asugi.sanita.fvg.it) |
| **FRIULI-VENEZIA GIULIA** | ASUGI – Centro Vaccinale | Grado | Via Buonarroti 10 | tel. 0481 487518  – 86 | [vaccinazionimn@asugi.sanita.f](mailto:vaccinazionimn@asugi.sanita.fvg.it) [vg.it](mailto:vaccinazionimn@asugi.sanita.fvg.it) |

| **FRIULI-VENEZIA GIULIA** | ASUFC - Latisana | Latisana | Via Sabbionera 45 | tel 0431 529926 | [ig-latisana@asufc.sanita.fvg.it](mailto:ig-latisana@asufc.sanita.fvg.it) |
| --- | --- | --- | --- | --- | --- |
| **FRIULI-VENEZIA**  **GIULIA** | ASUGI – Centro  Vaccinale | Monfalcone | Via Galvani 1 –  Ospedale San Polo | tel 0481 487518  – 86 | [vaccinazionimn@asugi.sanita.f](mailto:vaccinazionimn@asugi.sanita.fvg.it)  [vg.it](mailto:vaccinazionimn@asugi.sanita.fvg.it) |
| **FRIULI-VENEZIA GIULIA** | ASUFC - Palmanova | Palmanova | Via Molin 21 | tel 0432 921983 | [uff-ig-](mailto:uff-ig-palmanova@asufc.sanita.fvg.it) [palmanova@asufc.sanita.fvg.it](mailto:uff-ig-palmanova@asufc.sanita.fvg.it) |
| **FRIULI-VENEZIA GIULIA** | ASFO - Centro Vaccinale | Pordenone | Via Montereale, 32/A | tel 0434 1923200 | [prevenzione.pn@asfo.sanita.fv](mailto:prevenzione.pn@asfo.sanita.fvg.it) [g.it](mailto:prevenzione.pn@asfo.sanita.fvg.it) |
| **FRIULI-VENEZIA**  **GIULIA** | ASUFC - San Daniele  del Friuli | San Daniele del  Friuli | Via Trento e Trieste  4 | tel. 0432 949882 | [segr.dip@asufc.sanita.fvg.it](mailto:segr.dip@asufc.sanita.fvg.it) |
| **FRIULI-VENEZIA GIULIA** | ASUFC - Tarvisio | Tarvisio | Via V. Veneto 74 c/o Poliambulatorio | tel 0432 989338 | [segr.dip@asufc.sanita.fvg.it](mailto:segr.dip@asufc.sanita.fvg.it) |
| **FRIULI-VENEZIA GIULIA** | ASUFC - Tolmezzo | Tolmezzo | Via Morgagni 18 | tel 0433 488434 | [segr.dip@asufc.sanita.fvg.it](mailto:segr.dip@asufc.sanita.fvg.it) |
| **FRIULI-VENEZIA GIULIA** | ASUGI – Centro Vaccinale unico Trieste | Trieste | Via Paolo de’ Ralli 3 | tel 040 3997512 | [vaccinazioni@asugi.sanita.fvg.](mailto:vaccinazioni@asugi.sanita.fvg.it) [it](mailto:vaccinazioni@asugi.sanita.fvg.it) |
| **FRIULI-VENEZIA GIULIA** | USMAF SASN di  Veneto, Friuli Venezia Giulia e Trentino Alto Adige - TRIESTE | Trieste | Molo Fratelli Bandiera 1/1 | tel 040/303923; 06 59944817 | [usma.trieste@sanita.it](mailto:usma.trieste@sanita.it) |
| **FRIULI-VENEZIA GIULIA** | ASUFC - Udine | Udine | via Chiusaforte 2 | tel 0432/553225 | [medicina.viaggi@asufc.sanita.f](mailto:medicina.viaggi@asufc.sanita.fvg.it) [vg.it](mailto:medicina.viaggi@asufc.sanita.fvg.it) |
| **LAZIO** | Asl RM 4 - Bracciano | Bracciano (RM) | L.go dell'Ospedale Vecchio 8  00052 Bracciano (RM) | 800539762  lun - ven ore 9.00  - 13.00 | [infovaccini@aslroma4.it](mailto:infovaccini@aslroma4.it) |
| **LAZIO** | USMAF-SANS Lazio,  Marche, Umbria, Abruzzo e Molise - Unità  Territoriale di Ciampino | Ciampino (RM) | Aeroporto Ciampino  G.B. Pastine Via Appia Nuova 1651  00040 Ciampino RM | 06 65959420 | [usma.ciampino@sanita.it](mailto:usma.ciampino@sanita.it) |
| **LAZIO** | USMAF-SANS Lazio,  Marche, Umbria, Abruzzo e Molise- Unità Territoriale di Civitavecchia | Civitavecchia (RM) | Largo Plebiscito 4  00053 Civitavecchia (RM) | 06 59944837 | [usma.civitavecchia@sanita.it](mailto:usma.civitavecchia@sanita.it) |
| **LAZIO** | Asl RM 4 -  Civitavecchia | Civitavecchia (RM) | Piazza G. Verdi, 1  00053 Civitavecchia (RM) | 800539762  lun - ven ore 9.00  - 13.00 | [infovaccini@aslroma4.it](mailto:infovaccini@aslroma4.it) |
| **LAZIO** | ASL RM 5 - Medicina del turismo e del viaggiatore | Colleferro (RM) | Via degli Esplosivi 9 00034 Colleferro (RM) | 06 97097535 -  685 | [igienepubblica@aslroma5.it](mailto:igienepubblica@aslroma5.it) |
| **LAZIO** | Asl RM 4 - Fiano Romano | Fiano Romano (RM) | Via Capocroce 3  00065 Fiano  Romano | 800539762  lun - ven ore 9.00  - 13.01 | [infovaccini@aslroma4.it](mailto:infovaccini@aslroma4.it) |
| **LAZIO** | USMAF-SANS Lazio,  Marche, Umbria, Abruzzo e Molise- Unità Territoriale di Fiumicino | Fiumicino (RM) | Aeroporto Leonardo da Vinci - Torre Uffici 1  00054 Fiumicino (RM) | 06 65953251 | [usma.fiumicino@sanita.it](mailto:usma.fiumicino@sanita.it) |
| **LAZIO** | Asl FR - Vaccinazioni Internazionali | Frosinone | UOC Coordinamento Attività Vaccinali, Via A. Fabi, snc, Frosinone, Palazzina  N, Piano Terra | 07758822126  07758822128  07758822416 | [vaccinazioni.internazionali@as](mailto:vaccinazioni.internazionali@aslfrosinone.it) [lfrosinone.it](mailto:vaccinazioni.internazionali@aslfrosinone.it) |
| **LAZIO** | ASL RM 6 -  Vaccinazioni internazionali | Grottaferrata (RM | Viale San Nilo 4 00046 Grottaferrata (RM) | 06 93274124/5  lunedì-venerdì 8.30-11.30 |  |
| **LAZIO** | Asl LT - Centro di Medicina dei Viaggi c/o  C.C. Le Corbusier | Latina | c/o C.C. Le Corbusier - V.le Le Corbusier snc scala C, 2° piano  04100 Latina | 0773 6556850 | [vaccinazionidipprev@ausl.lati](mailto:vaccinazionidipprev@ausl.latina.it) [na.it](mailto:vaccinazionidipprev@ausl.latina.it) |
| **LAZIO** | Asl RM 3 Centro Vaccinale Casa della  salute | Ostia (RM) | Lung.mare Paolo Toscanelli 230  00121 Roma | 800605040 | [vaccinazioni@aslroma3.it](mailto:vaccinazioni@aslroma3.it) |
| **LAZIO** | ASL RI - Ambulatorio Medicina del Turismo - UOSD Malattie Infettive  - Ospedale S. Camillo de Lellis | Rieti | Ospedale S.Camillo de Lellis - Viale Kennedy - 02100 Rieti | 0746 278402  0746 278255 | [m.infettive@asl.rieti.it](mailto:m.infettive@asl.rieti.it) |

| **LAZIO** | ASL RI - Servizio Igiene e Sanità Pubblica - Centro Vaccinazioni Adulti | Rieti | Via Delle Ortensie n.28  02100 Rieti | 0746 278614  0746 279864  0746 279846 | [vaccinazioni.adulti@asl.rieti.it](mailto:vaccinazioni.adulti@asl.rieti.it) |
| --- | --- | --- | --- | --- | --- |
| **LAZIO** | Ambulatorio di “Occupational Health and Travel Clinic” c/o  Policlinico universitario "A. Gemelli"– Dip.to  Sanità Pubblica – sez. Medicina del Lavoro | Roma | L.go Agostino Gemelli,8 00168 Roma | 06 30157870  06 30157087 |  |
| **LAZIO** | FAO | Roma | solo per il personale dipendente |  |  |
| **LAZIO** | Ministero della Difesa | Roma | solo per il personale dipendente |  |  |
| **LAZIO** | ASL RM 2 - Centro Vaccinale Eucalipti | Roma | Via degli Eucalipti 14  00172 Roma | 06 51006666 | [centrovaccinale.d5@aslroma2.](mailto:centrovaccinale.d5@aslroma2.it) [it](mailto:centrovaccinale.d5@aslroma2.it) |
| **LAZIO** | Asl RM 3 – Centro Vaccinale Ozanam | Roma | Via F. Ozanam 126 00152 Roma | 800605040 | [vaccinazioni@aslroma3.it](mailto:vaccinazioni@aslroma3.it) |
| **LAZIO** | Poliambulatorio LIFEBRAIN LAZIO S.r.L. | Roma | Via Luigi Rizzo, 96 00136 Roma | 06 39730017 | [laboratorioaima@cerbahealthc](mailto:laboratorioaima@cerbahealthcare.it) [are.it](mailto:laboratorioaima@cerbahealthcare.it) |
| **LAZIO** | Asl RM 1 Centro  Vaccinazioni pediatriche e internazionali | Roma | Via Plinio, 31  00193 Roma | 06 68356087  06 68355317  06 68354031 | [medicinaviaggi@aslroma1.it](mailto:medicinaviaggi@aslroma1.it) |
| **LAZIO** | Istituto Nazionale per le Malattie Infettive (INMI)  L. Spallanzani - IRCCS | Roma | Via Portuense 292  00149 Roma | 06  55170232/237/39  3 |  |
| **LAZIO** | Asl RM 2 – Centro Vaccinazioni Internazionali San  Nemesio | Roma | Via San Nemesio 21 00145 Roma | 06 51006666 | [centrivaccinali.d8@aslroma2.it](mailto:centrivaccinali.d8@aslroma2.it) |
| **LAZIO** | Presidio sanitario ETIMEDICA S.r.l. | Roma | Viale degli Ammiragli, 67  00136 Roma | 06 99330428 | [info@etimedica.it](mailto:info@etimedica.it) |
| **LAZIO** | Ospedale Pediatrico Bambino Gesù - U.O. Immunologia Clinica e Vaccinologia | Roma | Viale di San Paolo 15  00146 Roma | CUP: 06 68181;  UO Immunologia e Vaccinologia: 06 68592372  Lun-mer-ven:  12.30 - 14.30 | [infovaccini@opbg.net](mailto:infovaccini@opbg.net) |
| **LAZIO** | USMAF-SANS Lazio,  Marche, Umbria, Abruzzo e Molise- Unità  Territoriale di Roma | Roma | Viale Giorgio Ribotta n° 5  00144 Roma | 06 59943727 | [usma.roma@sanita.it](mailto:usma.roma@sanita.it) |
| **LAZIO** | Istituto Superiore di Sanità | Roma | solo per il personale dipendente |  |  |
| **LAZIO** | Ambulatorio di Medicina dei Viaggi - Istituto Nazionale per la Promozione della salute delle popolazioni Migranti e per il contrasto delle malattie  della Povertà (INMP) | Roma | Via delle Fratte di Trastevere, 52 00153 Roma | CUP 06  58558503  lun-ven. 9.00/12.00;  06 58558205/206 | [segdirsanitaria@inmp.it](mailto:segdirsanitaria@inmp.it) |
| **LAZIO** | Asl Viterbo - Centro Medicina del Viaggiatore | Viterbo | Via Enrico Fermi n.15  Poliambulatorio I piano  stanza n.128 01100 Viterbo | 0761 236703 | [servizio.sisp@asl.vt.it](mailto:servizio.sisp@asl.vt.it) |
| **LIGURIA** | Ambulatorio di Medicina dei Viaggiatori | Chiavari (GE) | Corso Dante 163 | tel 0185 329023 -  37 | [ip.segreteria@asl4.liguria.it](mailto:ip.segreteria@asl4.liguria.it)  [certificati.vaccinali@asl4.ligur](mailto:certificati.vaccinali@asl4.liguria.it) [ia.it](mailto:certificati.vaccinali@asl4.liguria.it) |
| **LIGURIA** | Ambulatorio Medicina  Viaggi ASL 3 | Genova | Via Operai 80 | tel 010 8497033 | [vaccinazioni@asl3.liguria.it](mailto:vaccinazioni@asl3.liguria.it) |
| **LIGURIA** | USMAF SASN di  Liguria | Genova | Ponte Andrea Doria snc, Genova Porto | tel 06 59944787 | [usma.genova@sanita.it](mailto:usma.genova@sanita.it) |

| **LIGURIA** | USMAF SASN di  Liguria - Unità Territoriale di Genova | Genova | Via Cantore 3 | tel 06 59944787 | [usma.genova@sanita.it](mailto:usma.genova@sanita.it) |
| --- | --- | --- | --- | --- | --- |
| **LIGURIA** | Ambulatorio Medicina Viaggi ASL 3 Genovese | Genova | Via Archimede 30-A | tel 010 8494964  – 8497033 | [vaccinazioni@asl3.liguria.it](mailto:vaccinazioni@asl3.liguria.it) |
| **LIGURIA** | Ambulatorio Vaccinazioni Palasalute | Imperia | Via Lorenzo Acquarone 9 | tel 0183 537 635 | [im.vaccinazioni@asl1.liguria.it](mailto:im.vaccinazioni@asl1.liguria.it) |
| **LIGURIA** | USMAF SASN di  Liguria- Unità territoriale di Imperia | Imperia | Viale Matteotti, 177 | tel 06 59944717 | [usma.imperia@sanita.it](mailto:usma.imperia@sanita.it) |
| **LIGURIA** | USMAF SASN di  Liguria - Unità Territoriale di La Spezia | La Spezia | Piazza Europa 11, 4° Piano | tel 06 5994 4716 | [usma.laspezia@sanita.it](mailto:usma.laspezia@sanita.it) |
| **LIGURIA** | Ambulatorio Medicina Viaggi USL 5 - Casa della salute - Spezzino | Sarzana (SP) | Via A. Paci 1 | tel 0187 604898 -  0187 604236 | [medicina.viaggi@asl5.liguria.i](mailto:medicina.viaggi@asl5.liguria.it) [t](mailto:medicina.viaggi@asl5.liguria.it) |
| **LIGURIA** | USMAF SASN di  Liguria - Unità territoriale di Savona | Savona | Lungomare Matteotti, 5 | tel 06 5994 4803 | [usma.savona@sanita.it](mailto:usma.savona@sanita.it) |
| **LIGURIA** | Centro di Medicina dei Viaggiatori U.O.I.S.P. | Savona | Via Collodi 13 | tel 019 8405901 | [vaccinazioni.savona@asl2.ligu](mailto:vaccinazioni.savona@asl2.liguria.it) [ria.it](mailto:vaccinazioni.savona@asl2.liguria.it) |
| **LOMBARDIA** | ATS BERGAMO | Bergamo | Via Borgo Palazzo 130 | tel 035 2676500/522 | [presstbg.vaccinazioni@asst-](mailto:presstbg.vaccinazioni@asst-pg23.it) [pg23.it](mailto:presstbg.vaccinazioni@asst-pg23.it) |
| **LOMBARDIA** | USMAF SASN di  Lombardia, Piemonte e Valle d' Aosta – Ufficio Territoriale di Orio al Serio | Bergamo - Orio al Serio | Via Aeroporto 13 c/o Aeroporto Caravaggio | tel 06 5994 4782 | [usma.bergamo@sanita.it](mailto:usma.bergamo@sanita.it) |
| **LOMBARDIA** | CITTA’ METROPOLITANA DI  MILANO - POT Bollate | Bollate (MI) | Via Piave 20 | tel numero verde 800.671.671,  giorni lavorativi 9,00-15,00 |  |
| **LOMBARDIA** | ATS MONTAGNA -  Ambulatorio vaccinazioni Bormio | Bormio (SO) | Via Agoi 8 | tel 0342 909155 | [vaccinazioni.bo@asst-val.it](mailto:vaccinazioni.bo@asst-val.it) |
| **LOMBARDIA** | ATS BRESCIA -  Bovezzo | Bovezzo (BS) | Via Vittorio Veneto 17 | tel 030/3537122  prenotazione lun-  ven 8,30-16,30 |  |
| **LOMBARDIA** | ATS MONTAGNA -  Ambulatorio vaccinazioni Breno | Breno (BS) | Via Nissolina 2 | tel 800 270662  CUP (tasto 4);  0364 329369  (info) | [vaccinazioni@asst-](mailto:vaccinazioni@asst-valcamonica.it) [valcamonica.it](mailto:vaccinazioni@asst-valcamonica.it) |
| **LOMBARDIA** | ATS BRESCIA - Brescia | Brescia | Via Acerbi 8 | tel 030/3537122  prenotazione lun-  ven 8,30-16,30 |  |
| **LOMBARDIA** | ATS INSUBRIA - Cantù | Cantù (CO) | Via Domea 4 | tel 031/ 799223 | Portale vaccinazioni SITO ASST LARIANA |
| **LOMBARDIA** | ATS BRESCIA - Chiari | Chiari | Piazza Martiri della Libertà 25 | tel 030 7103019 | [vaccinazioni.chiari@asst-](mailto:vaccinazioni.chiari@asst-franciacorta.it) [franciacorta.it](mailto:vaccinazioni.chiari@asst-franciacorta.it) |
| **LOMBARDIA** | HUB vaccinale Italmark | Chiari (BS) | Via Brescia, 31 | tel 3336133754  mer. 08,30 -  12,15 (su  appuntamento) | [vaccinazioni.chiari@asst-](mailto:vaccinazioni.chiari@asst-franciacorta.it) [franciacorta.it](mailto:vaccinazioni.chiari@asst-franciacorta.it) |
| **LOMBARDIA** | ATS MONTAGNA -  Ambulatorio vaccinazioni Chiavenna | Chiavenna (SO) | Via Cereria, 4 | tel 0343 67330 | [vaccinazioni.ch@asst-val.it](mailto:vaccinazioni.ch@asst-val.it) |
| **LOMBARDIA** | ATS INSUBRIA - Como | Como | Via Napoleona 60 | tel 800 893526 | Portale vaccinazioni SITO  ASST LARIANA |
| **LOMBARDIA** | ATS VAL PADANA -  Poliambulatori ASST CREMONA | Crema (CR) | Largo U. Dossena 2 | tel 0373 280091 | [vaccinazioni@asst-crema.it](mailto:vaccinazioni@asst-crema.it) |
| **LOMBARDIA** | ATS VAL PADANA | Cremona | Via Dante, 134 | tel 0372/408616 | [vaccinazioni@asst-cremona.it](mailto:vaccinazioni@asst-cremona.it) |
| **LOMBARDIA** | Centro vaccinazioni  internazionali | Dalmine (BG) | Viale Betelli 2 | 035/378121 | [vaccinazioni.dalmine@asst-](mailto:vaccinazioni.dalmine@asst-bgovest.it)  [bgovest.it](mailto:vaccinazioni.dalmine@asst-bgovest.it) |
| **LOMBARDIA** | ATS MONTAGNA -  Ambulatorio vaccinazioni DARFO B.T. | Darfo Boario Terme (BS) | Via Cercovi 2 | tel 800 270662  CUP (tasto 4);  0364 329368  (info) | [vaccinazioni@asst-](mailto:vaccinazioni@asst-valcamonica.it) [valcamonica.it](mailto:vaccinazioni@asst-valcamonica.it) |
| **LOMBARDIA** | ATS BRESCIA -  Desenzano del Garda | Desenzano del Garda  (BS) | Via Adua 4 | tel 030 9116727;  per prenotazioni | [vacccinazioni.desenzano@asst](mailto:vacccinazioni.desenzano@asst-garda.it)  [-garda.it](mailto:vacccinazioni.desenzano@asst-garda.it) |

|  |  |  |  | 030 9037555 (CUP) |  |
| --- | --- | --- | --- | --- | --- |
| **LOMBARDIA** | ATS BRIANZA - Desio | Desio (MB) | Via Mazzini 1 c/o  Ospedale Desio |  | [desio.vaccinazioni@asst-](mailto:desio.vaccinazioni@asst-brianza.it)  [brianza.it](mailto:desio.vaccinazioni@asst-brianza.it) |
| **LOMBARDIA** | AMBULATORIO VACCINAZIONI DONGO | Dongo (CO) | Via Falck, 3 | tel 0344 973519 | [vaccinazioni.do@asst-val.it](mailto:vaccinazioni.do@asst-val.it) |
| **LOMBARDIA** | ATS MONTAGNA -  Ambulatorio vaccinazioni Edolo | Edolo (BS) | Piazza Donatori Sangue 1 | tel 800 270662  (tasto 4) 0364  329368 (info) | [vaccinazioni@asst-](mailto:vaccinazioni@asst-valcamonica.it) [valcamonica.it](mailto:vaccinazioni@asst-valcamonica.it) |
| **LOMBARDIA** | ATS INSUBRIA - HUB  Gallarate | Gallarate (VA) | Viale Milano 153 | tel 0331/699168 | [vaccinazioni.viaggi@asst-](mailto:vaccinazioni.viaggi@asst-valleolona.it) [valleolona.it](mailto:vaccinazioni.viaggi@asst-valleolona.it) |
| **LOMBARDIA** | ATS BRESCIA -  Gavardo | Gavardo (BS) | Via A. Gosa, 74 | tel 030 9116 771;  per prenotazioni 030 9037555 (CUP) | [vaccinazioni.salo@asst-](mailto:vaccinazioni.salo@asst-garda.it) [garda.it](mailto:vaccinazioni.salo@asst-garda.it) |
| **LOMBARDIA** | Casa della Comunità Gazzaniga | Gazzaniga (BG) | Via A. Manzoni, 130 | tel 035/3601111 |  |
| **LOMBARDIA** | ATS BRESCIA -  Gussago | Gussago (BS) | Via Richiedei 8/B | tel 030/3537122  prenotazione lun- ven 8,30-16,30 |  |
| **LOMBARDIA** | ATS BRIANZA - Lecco | Lecco | Via Tubi 43 | tel 0341/253900  (lunedì-venerdì 9.00-  12.00/13.30-  15.30) | [cuv.lecco@asst-lecco.it](mailto:cuv.lecco@asst-lecco.it) |
| **LOMBARDIA** | CITTÀ METROPOLITANA DI  MILANO - Centro vaccinale Legnano | Legnano (MI) | Via Canazza 2 |  | [vaccinazioni.areapagamento@](mailto:vaccinazioni.areapagamento@asst-ovestmi.it) [asst-ovestmi.it](mailto:vaccinazioni.areapagamento@asst-ovestmi.it) |
| **LOMBARDIA** | ATS BRESCIA - Leno | Leno (BS) | Piazza Donatori Sangue 1 | tel 030 9116408;  prenotazioni 030  9037555 (CUP) | [vaccinazioni.leno@asst-](mailto:vaccinazioni.leno@asst-garda.it) [garda.it](mailto:vaccinazioni.leno@asst-garda.it) |
| **LOMBARDIA** | Ambulatorio vaccinazioni Livigno | Livigno (SO) | Via Freita, 1521/B | tel 0342 909155 | [vaccinazioni.bo@asst-val.it](mailto:vaccinazioni.bo@asst-val.it) |
| **LOMBARDIA** | CITTÀ METROPOLITANA DI  MILANO - Lodi | Lodi | Via Bassi 1 | tel 0371 372492 |  |
| **LOMBARDIA** | Centro vaccinale CAVOK Medical Center | Lonate Pozzolo (VA) | Via del gregge, 100 | tel 0331 116  0008 |  |
| **LOMBARDIA** | CITTÀ METROPOLITANA DI  MILANO - Centro vaccinale Magenta | Magenta (MI) | Via al Donatore di Sangue 50 |  | [vaccinazioni.areapagamento@](mailto:vaccinazioni.areapagamento@asst-ovestmi.it) [asst-ovestmi.it](mailto:vaccinazioni.areapagamento@asst-ovestmi.it) |
| **LOMBARDIA** | ATS VAL PADANA  Ambulatorio Viaggiatori Internazionali | Mantova | Via dei Toscani 1 – Palazzina 10 | tel 0376/435789,  prenotazione lun- ven 11,00-12,30 | [polovaccinale.mantova@asst-](mailto:polovaccinale.mantova@asst-mantova.it) [mantova.it](mailto:polovaccinale.mantova@asst-mantova.it) |
| **LOMBARDIA** | Ambulatorio vaccinazioni internazionali Martinengo | Martinengo (BG) | Piazza maggiore, 11 | tel 0363/919219 | [vaccinazioni.romano@asst-](mailto:vaccinazioni.romano@asst-bgovest.it) [bgovest.it](mailto:vaccinazioni.romano@asst-bgovest.it) |
| **LOMBARDIA** | CITTÀ METROPOLITANA DI  MILANO - Melzo | Melzo (MI) | Via Gavazzi, 100 | tel 02/98118555  su appuntamento telefonico giov. 9,00-12,00 |  |
| **LOMBARDIA** | ATS INSUBRIA -  Menaggio | Menaggio (CO) | Via Casartelli 7 | tel 0344/33217 | [vaccinazioni.menaggio@asst-](mailto:vaccinazioni.menaggio@asst-lariana.it) [lariana.it](mailto:vaccinazioni.menaggio@asst-lariana.it) |
| **LOMBARDIA** | ATS BRIANZA -  Merate | Merate (LC) | Largo Mandic 1 | tel 0341/253900  (lunedì-venerdì 9.00-  12.00/13.30-  15.30) | [cuv.lecco@asst-lecco.it](mailto:cuv.lecco@asst-lecco.it) |
| **LOMBARDIA** | CITTÀ METROPOLITANA DI  MILANO | Milano | Via Statuto 5 | tel 800 638638,  (02 999599 da  cellulare) | [vaccinazioni.internazionali@as](mailto:vaccinazioni.internazionali@asst-fbf-sacco.it) [st-fbf-sacco.it](mailto:vaccinazioni.internazionali@asst-fbf-sacco.it) |
| **LOMBARDIA** | CITTÀ METROPOLITANA DI  MILANO Centro Polispecialistico Pacini s.r.l. | Milano | Via Giovanni Pacini 15 | tel 02 2361230 | [info@polispecialisticopacini.it](mailto:info@polispecialisticopacini.it) |

| **LOMBARDIA** | CITTÀ METROPOLITANA DI  MILANO Centro Diagnostico Italiano | Milano | Via Saint Bon 20 | tel 02/48317333- 7444 -  02/483171  (centralino); Prenotazioni Call Center 02 -  48317333 |  |
| --- | --- | --- | --- | --- | --- |
| **LOMBARDIA** | CITTÀ METROPOLITANA DI  MILANO Ambulatorio di medicina tropicale, dei viaggi e delle migrazioni – Centro San Luigi distaccamento di San  Raffaele Turro | Milano | Via Stamira D’Ancona 20 | tel 02/26437970  - 02/26434180  Mer e Ven 13,00  - 17,00;  Informazioni 02/26434979 | [medicinadeiviaggi@hsr.it](mailto:medicinadeiviaggi@hsr.it) |
| **LOMBARDIA** | CITTÀ METROPOLITANA DI  MILANO Società e Salute srl – Centro  Medico Sant’Agostino | Milano | Piazza Sant’Agostino | tel 02 89701701 | [informazioni@cmsantagostino.](mailto:informazioni@cmsantagostino.it) [it](mailto:informazioni@cmsantagostino.it) |
| **LOMBARDIA** | USMAF SASN di  Lombardia, Piemonte e Valle d' Aosta – Ufficio Territoriale di Linate | Milano | Viale Enrico Forlanini c/o Aeroporto Forlanini | tel 06 5994 4779 | [usma.linate@sanita.it](mailto:usma.linate@sanita.it) |
| **LOMBARDIA** | ATS BRESCIA -  Montichiari | Montichiari (BS) | Via Falcone 18 | tel 030/9116248;  CUP per  prenotazione 030/9037555 | [vaccinazioni.montichiari@asst](mailto:vaccinazioni.montichiari@asst-garda.it)  [-garda.it](mailto:vaccinazioni.montichiari@asst-garda.it) |
| **LOMBARDIA** | ATS BRIANZA - Monza | Monza | Via De Amicis 17 | Coordinatore 039/2335353; CCR 800638638  /02 999599 | [monza.vaccinazioni@asst-](mailto:monza.vaccinazioni@asst-monza.it) [monza.it](mailto:monza.vaccinazioni@asst-monza.it) |
| **LOMBARDIA** | ATS MONTAGNA -  Ambulatorio vaccinazioni Morbegno | Morbegno (SO) | Via G.B. Martinelli 13 | tel 0342 643271 | [vaccinazioni.mo@asst-val.it](mailto:vaccinazioni.mo@asst-val.it) |
| **LOMBARDIA** | ATS BERGAMO - Pavia | Pavia | V.le Indipendenza 5 | tel 0382 1958412 | [vaccinazioni_pavia@asst-](mailto:vaccinazioni_pavia@asst-pavia.it) [pavia.it](mailto:vaccinazioni_pavia@asst-pavia.it) |
| **LOMBARDIA** | Centro vaccinale di Ponte Lambro | Ponte Lambro (CO) | Via Verdi, 2 | tel 031/6337906 | [vaccinazioni.distrettodierba@a](mailto:vaccinazioni.distrettodierba@asst-lariana.it) [sst-lariana.it](mailto:vaccinazioni.distrettodierba@asst-lariana.it) |
| **LOMBARDIA** | Ambulatorio vaccinazioni internazionali Ponte San Pietro | Ponte San Pietro (BG) | Via Caironi, 7 | tel 035/603256 | [vaccinazioni.pontesanpietro@a](mailto:vaccinazioni.pontesanpietro@asst-bgovest.it) [sst-bgovest.it](mailto:vaccinazioni.pontesanpietro@asst-bgovest.it) |
| **LOMBARDIA** | ATS BRESCIA | Rezzato (BS) | Via Kennedy 115 | tel 030/3537122  prenotazione lun- ven 8,30-16,30 |  |
| **LOMBARDIA** | CITTA’ METROPOLITANA DI  MILANO - Rozzano | Rozzano (MI) | Via Magnolie 2 | tel 02 82456011 -  12 |  |
| **LOMBARDIA** | ATS INSUBRIA -  Saronno | Saronno (VA) | Via Fiume 12 | tel 02 9613914 -  5 (lun-ven 11,00-  12,00) |  |
| **LOMBARDIA** | ATS INSUBRIA - Sesto  Calende | Sesto Calende (VA) | L.go Cardinale Dell'Acqua 1 | tel 0331 965028  (lun-ven 10,00-  11,00) |  |
| **LOMBARDIA** | CITTA’ METROPOLITANA DI  MILANO - Sesto San Giovanni - Distretto 6 | Sesto S. Giovanni (MI) | Via Oslavia 1 | tel 0257994458 |  |
| **LOMBARDIA** | ATS BERGAMO -  ambulatorio vaccinazioni Sondrio | Sondrio | Via Stelvio 32/A, c/o Condominio 3 Stelle | tel 0342 555423 | [vaccinazioni.so@asst-val.it](mailto:vaccinazioni.so@asst-val.it) |
| **LOMBARDIA** | ATS MONTAGNA | Tirano (SO) | Via Pedrotti, 57 | tel 0342 707340 | [vaccinazioni.ti@asst-val.it](mailto:vaccinazioni.ti@asst-val.it) |
| **LOMBARDIA** | ATS INSUBRIA -  Tradate | Tradate (VA) | Via Gorizia 42 | tel 0331 815411 |  |
| **LOMBARDIA** | ATS BERGAMO -  Centro vaccinale viaggiatori internazionali TRESCORE | Trescore Balneario (BG) | Via Ospedale 38 |  | [vaccinazioni.trescore@asst-](mailto:vaccinazioni.trescore@asst-bergamoest.it) [bergamoest.it](mailto:vaccinazioni.trescore@asst-bergamoest.it) |

| **LOMBARDIA** | ATS BERGAMO Centro vaccinazioni internazionali | Treviglio (BG) | Piazzale Ospedale L. Meneguzzo, 1 | tel 0363/590900 | [vaccinazioni.treviglio@asst-](mailto:vaccinazioni.treviglio@asst-bgovest.it) [bgovest.it](mailto:vaccinazioni.treviglio@asst-bgovest.it) |
| --- | --- | --- | --- | --- | --- |
| **LOMBARDIA** | ATS BRIANZA -  Usmate | Usmate (MB) | Via Roma 85 | tel 039/6654981,  il mercoledì dalle 13.30-15.30 (solo  per informazioni) | [profilassi.viaggiatori@asst-](mailto:profilassi.viaggiatori@asst-brianza.it) [brianza.it](mailto:profilassi.viaggiatori@asst-brianza.it) ; link per richiesta appuntamento: [https://sv.asst-](https://sv.asst-brianza.it/) [brianza.it](https://sv.asst-brianza.it/) |
| **LOMBARDIA** | ATS INSUBRIA -  Varese | Varese | Via O. Rossi 9 | tel 0332 277392,  (prenotazione mer 10,00-12,00) | [vaccinazioni.varese@asst-](mailto:vaccinazioni.varese@asst-settelaghi.it) [settelaghi.it](mailto:vaccinazioni.varese@asst-settelaghi.it) |
| **LOMBARDIA** | USMAF SASN di  Lombardia, Piemonte e Valle d' Aosta – Ufficio Territoriale di Milano Malpensa | Varese | Aereoporto di Malpensa – Terminal 2 21010 Ferno (VA) | tel 06 5994 4793 | [usma.malpensa.ambulatorio@s](mailto:usma.malpensa.ambulatorio@sanita.it) [anita.it](mailto:usma.malpensa.ambulatorio@sanita.it) ; [usma.varese@sanita.it](mailto:usma.varese@sanita.it) |
| **LOMBARDIA** | ATS BERGAMO -  Zogno | Zogno (BG) | Piazza Bortolo Belotti 1-3 | tel 0345 545844-  46 | [presstvalli.vaccinazioni@asst-](mailto:presstvalli.vaccinazioni@asst-pg23.it) [pg23.it](mailto:presstvalli.vaccinazioni@asst-pg23.it) |
| **MARCHE** | USMAF SASN di Lazio, Marche, Umbria, Abruzzo e Molise - Unità Territoriale di Ancona | Ancona | Banchina Nazario Sauro 60121 | tel 06 5994 4790 | email: [usma.ancona@sanita.it](mailto:usma.ancona@sanita.it) |
| **MARCHE** | ASUR – Area Vasta 2 - Centro Medicina del Viaggiatore | Ancona | Via Cristoforo Colombo 106 | tel 071 8705547 -  52 |  |
| **MARCHE** | ASUR - Area Vasta 4 | Ascoli Piceno | Viale Marcello Federici (EX GIL) | tel 0736 358034 |  |
| **MARCHE** | ASUR Area Vasta 3 | Civitanova Marche | Via Ginocchi | tel 0733- 823712 |  |
| **MARCHE** | ASUR Area Vasta 2 | Fabriano | Via F. Turati 51 | tel 0732 634107 |  |
| **MARCHE** | ASUR Area Vasta 1 -  U.O.C. ISP Prevenzione Malattie Infettive | Fano | Via Borsellino 4 | tel 0721 868986  (amb) 800  098798 (CUP) |  |
| **MARCHE** | Centro Medico Polispecialistico BIOS | Fano | Via del Risorgimento 6/A | tel 0721 801617 |  |
| **MARCHE** | ASUR Area Vasta 4 | Fermo | Via Zeppilli 22A | tel 0734 6253381 |  |
| **MARCHE** | ASUR Area Vasta 2 | Jesi | Via Guerri 9 | tel 0731 534672 |  |
| **MARCHE** | ASUR Area Vasta 3 | Macerata | Via Annibali 31/L - Piediripa | tel 0733 2572679 |  |
| **MARCHE** | ASUR Area Vasta 1 -  U.O.C. ISP Prevenzione Malattie Infettive | Pesaro | Via F. Nitti 3 | tel 0721 424415-  21 |  |
| **MARCHE** | ASUR Area Vasta 4 | San Benedetto del  Tronto | Piazza Nardone 19 | tel 0735 793663 |  |
| **MARCHE** | ASUR Area Vasta 3 c/o Ospedale Bartolomeo Eustachio | San Severino Marche | Via del Glorioso 8 | tel. 0733 642302 |  |
| **MARCHE** | Senigallia – ASUR Area Vasta 2 | Senigallia | Via Po 13 | tel 071 79092325 |  |
| **MARCHE** | ASUR Area Vasta 1 -  U.O.C. ISP Prevenzione Malattie Infettive | Urbino | Via Comandino 21 | tel 0722 301702-  16 |  |
| **MARCHE** | Centro Medico Polispecialistico BIOS | Vallefoglia | Via Giacometti 36 | tel 0721 472206 |  |
| **MOLISE** | U.O.C. Igiene e Sanità Pubblica | Campobasso | Via Toscana 77 | tel 0874 409127  – 6 fax: 0874  313131 |  |
| **MOLISE** | U.O.C. Igiene e Sanità Pubblica | Isernia | Largo Cappuccini 1 | tel 0865 442557,  0865 4425580  fax: 0865  4425571 |  |
| **MOLISE** | U.O.C. Igiene e Sanità Pubblica | Termoli | Via del Molinello 1 | tel 0875 7159703  fax: 0875  7159683 |  |
| **P.A. BOLZANO** | Azienda sanitaria della Provincia di Bolzano | Bolzano | Via Amba Alagi 33 | tel 0471 909264 |  |
| **P.A. BOLZANO** | Azienda sanitaria dell’Alto Adige | Bressanone | Via Dante 51 | tel 0472 250900 |  |
| **P.A. BOLZANO** | Azienda sanitaria  dell’Alto Adige | Brunico | Vicolo dei Frati 3 | tel 0472 250900 |  |

| **P.A. BOLZANO** | Azienda sanitaria della Provincia di Bolzano | Merano | Via Goethe 7 | tel 0473 251828 -  6 |  |
| --- | --- | --- | --- | --- | --- |
| **P.A. TRENTO** | UO Cure primarie, Igiene pubblica Vallagarina e Altipiani  Cimbri | Rovereto | Piazza A. Leoni 11/A | Tel 0464 403704 |  |
| **P.A. TRENTO** | Centro Servizi Sanitari | Trento | Viale Verona | tel 0461 904601 |  |
| **PIEMONTE** | ASL CUNEO 2 | Alba | Via Vida 10 | tel 0173 316619 | [vaccinazioni.alba@aslcn2.it](mailto:vaccinazioni.alba@aslcn2.it) |
| **PIEMONTE** | ASL ALESSANDRIA | Alessandria | Via Venezia 6 | tel 0131 306966 | [medviaggi.alessandria@aslal.it](mailto:medviaggi.alessandria@aslal.it) |
| **PIEMONTE** | ASL ASTI | Asti | Via Conte Verde 125 | tel 0141 484943  – 53 | [simi@asl.at.it](mailto:simi@asl.at.it) |
| **PIEMONTE** | ASL BIELLA | Biella | Via Don Sturzo 20 | tel 015 15159261 | [vaccinazioni@aslbi.piemonte.i](mailto:vaccinazioni@aslbi.piemonte.it) [t](mailto:vaccinazioni@aslbi.piemonte.it) |
| **PIEMONTE** | ASL TORINO 4 | Borgaro Torinese | Via SS. Cosma e  Damiano 1 | tel 011 4211606 | [sispvaccinazioni.cirie@aslto4.](mailto:sispvaccinazioni.cirie@aslto4.piemonte.it)  [piemonte.it](mailto:sispvaccinazioni.cirie@aslto4.piemonte.it) |
| **PIEMONTE** | ASL VERCELLI | Borgosesia (VC) – c/o Osp. SS Pietro e Paolo | Via A.F. Ilorini Mo, 20 | tel 0163 426722 | [vaccinazioni@aslvc.piemonte.i](mailto:vaccinazioni@aslvc.piemonte.it) [t](mailto:vaccinazioni@aslvc.piemonte.it) |
| **PIEMONTE** | ASL TORINO 5 | Carmagnola | Via Avv. Ferrero 26 | tel 011 6930500 | [vaccinazioni@aslto5.piemonte.](mailto:vaccinazioni@aslto5.piemonte.it) [it](mailto:vaccinazioni@aslto5.piemonte.it) |
| **PIEMONTE** | ASL ALESSANDRIA | Casale Monferrato | Via Palestro 41 | tel 0142  434531/49 | [medviaggi.alessandria@aslal.it](mailto:medviaggi.alessandria@aslal.it) |
| **PIEMONTE** | ASL CUNEO 1 | Cuneo | C.so Francia 10 | tel 0171 078680 | [sisp@aslcn1.it](mailto:sisp@aslcn1.it) |
| **PIEMONTE** | ASL VERBANO- CUSIO-COSSOLA | Domodossola | Via Scapaccino 47 | tel 0324 491629 | [sisp.do@aslvco.it](mailto:sisp.do@aslvco.it) |
| **PIEMONTE** | ASL CUNEO 1 | Fossano | Via Lancimano 9 | tel 0171 078680 | [sisp@aslcn1.it](mailto:sisp@aslcn1.it) |
| **PIEMONTE** | ASL TORINO 4 | Ivrea | Via Aldisio 2 | tel 0125 414713 | [sispvaccinazioni.ivrea@aslto4.](mailto:sispvaccinazioni.ivrea@aslto4.piemonte.it) [piemonte.it](mailto:sispvaccinazioni.ivrea@aslto4.piemonte.it) |
| **PIEMONTE** | ASL CUNEO 1 | Mondovì – c/o Osp. Regina Montis Regalis | Via S. Rocchetto 99 | tel 0171 078680 | [sisp@aslcn1.it](mailto:sisp@aslcn1.it) |
| **PIEMONTE** | ASL NOVARA | Novara | Viale Roma 7A | tel 0321 374304 /  705 | [vaccinazioniadulti@asl.novara.](mailto:vaccinazioniadulti@asl.novara.it)  [it](mailto:vaccinazioniadulti@asl.novara.it) |
| **PIEMONTE** | ASL ALESSANDRIA | Novi Ligure | Via Papa Giovanni XXIII 1 | tel 0143 332638/39 | [medviaggi.alessandria@aslal.it](mailto:medviaggi.alessandria@aslal.it) |
| **PIEMONTE** | ASL VERBANO- CUSIO-COSSOLA | Omegna | Via IV Novembre 294 | tel 0323 868058 | [sisp.om@aslvco.it](mailto:sisp.om@aslvco.it) |
| **PIEMONTE** | ASL TORINO 3 | Pinerolo | Via Bignone 15/A | tel 800090088 | [sisp.vaccinazioni@aslto3.piem](mailto:sisp.vaccinazioni@aslto3.piemonte.it)  [onte.it](mailto:sisp.vaccinazioni@aslto3.piemonte.it) |
| **PIEMONTE** | ASL TORINO 3 | Rivoli | Via Balegno 6 | tel 800090088 | [sisp.vaccinazioni@aslto3.piem](mailto:sisp.vaccinazioni@aslto3.piemonte.it) [onte.it](mailto:sisp.vaccinazioni@aslto3.piemonte.it) |
| **PIEMONTE** | ASL CUNEO 1 | Saluzzo | Via del Follone 4 | tel 0171 078680 | [sisp@aslcn1.it](mailto:sisp@aslcn1.it) |
| **PIEMONTE** | ASL TORINO 4 | Settimo Torinese | Via Regio Parco 64 | tel 011 8212339/367 | [sispvaccinazioni.settimo@aslt](mailto:sispvaccinazioni.settimo@aslto4.piemonte.it) [o4.piemonte.it](mailto:sispvaccinazioni.settimo@aslto4.piemonte.it) |
| **PIEMONTE** | USMAF - SASN  Lombardia, Piemonte e Valle d'Aosta - Ufficio Territoriale di Torino | Torino | Strada Aeroporto 12 c/o Aeroporto S. Pertini - Caselle Torinese (TO) | tel 06/59948708; 0115676848 | [usma.torino@sanita.it](mailto:usma.torino@sanita.it) |
| **PIEMONTE** | Asl Città di Torino | Torino | Via Consolata 10 | tel 011 5663054 | [vaccinazioni@aslcittaditorino.i](mailto:vaccinazioni@aslcittaditorino.it) [t](mailto:vaccinazioni@aslcittaditorino.it) |
| **PIEMONTE** | Asl Città di Torino c/o Osp. Amedeo di Savoia | Torino | C.so Svizzera 164 | tel 011 4393803/3900 | [viaggi@aslcittaditorino.it](mailto:viaggi@aslcittaditorino.it) |
| **PIEMONTE** | ASL VERBANO- CUSIO-COSSOLA | Verbania | Viale S. Anna 83 | tel 0323 541458 | [sisp.vb@aslvco.it](mailto:sisp.vb@aslvco.it) |
| **PIEMONTE** | ASL VERCELLI | Vercelli | Largo Giusti 13 | tel 0161 593030 | [vaccinazioni@aslvc.piemonte.i](mailto:vaccinazioni@aslvc.piemonte.it) [t](mailto:vaccinazioni@aslvc.piemonte.it) |
| **PUGLIA** | ASL BAT - Centro di  medicina dei viaggi e delle migrazioni | Andria | Viale Trentino 79 | tel 0883 299513  – 502 | [direzione.sisp@aslbat.it](mailto:direzione.sisp@aslbat.it) |
| **PUGLIA** | USMAF SASN di  Puglia, Calabria e Basilicata - Unità Territoriale di Bari | Bari | C.so Antonio De Tullio 3 (interno Porto) | tel 06 5994 4806 | [usma.bari@sanita.it](mailto:usma.bari@sanita.it) |
| **PUGLIA** | ASL BARI –  Ambulatorio medicina dei viaggi e  delle migrazioni | Bari | Lungomare Starita 6 | tel 080 5842618 | [medicina.viaggi@asl.bari.it](mailto:medicina.viaggi@asl.bari.it) |

| **PUGLIA** | ASL BRINDISI –  Ambulatorio vaccinale | Brindisi | Piazza A. Di Summa 1 | tel 0831 510404 | [ufficiovaccinazioni.brindisi@a](mailto:ufficiovaccinazioni.brindisi@asl.brindisi.it) [sl.brindisi.it](mailto:ufficiovaccinazioni.brindisi@asl.brindisi.it) |
| --- | --- | --- | --- | --- | --- |
| **PUGLIA** | USMAF SASN di  Puglia, Calabria e Basilicata - Unità Territoriale di Brindisi | Brindisi | Stazione Marittima - V.le Regina Giovanna di Bulgaria  s.n. | tel 06 5994 4812 | [usma.brindisi@sanita.it](mailto:usma.brindisi@sanita.it) |
| **PUGLIA** | ASL TARANTO –  Ambulatorio vaccinale | Castellaneta | Via del Mercato c/o Osp. Vecchio (4°  piano) | tel 099 8496244 | [diprev.sisp.castellaneta@asl.tar](mailto:diprev.sisp.castellaneta@asl.taranto.it) [anto.it](mailto:diprev.sisp.castellaneta@asl.taranto.it) |
| **PUGLIA** | ASL FOGGIA –  Ambulatorio vaccinale | Cerignola | V.le G. Di Vittorio  21 | tel 338-4720432 | [ambulatoriocerignola@gmail.c](mailto:ambulatoriocerignola@gmail.com)  [om](mailto:ambulatoriocerignola@gmail.com) |
| **PUGLIA** | ASL BRINDISI –  Ambulatorio vaccinale | Fasano | Via Nazionale dei Trulli, 95 | tel 080 4390314 | [ufficiovaccinazioni.fasano@asl](mailto:ufficiovaccinazioni.fasano@asl.brindisi.it)  [.brindisi.it](mailto:ufficiovaccinazioni.fasano@asl.brindisi.it) |
| **PUGLIA** | ASL FOGGIA –  Ambulatorio vaccinale | Foggia | Piazza Pavoncelli 11 | Tel 0881 884304 | [uovaccinazionifg@aslfg.it](mailto:uovaccinazionifg@aslfg.it) |
| **PUGLIA** | ASL TARANTO –  Ambulatorio vaccinale | Grottaglie | Via Portelle delle  Ginestre ang. 1° Maggio | tel 099 860471 | [diprev.sisp.grottaglie@asl.tara](mailto:diprev.sisp.grottaglie@asl.taranto.it) [nto.it](mailto:diprev.sisp.grottaglie@asl.taranto.it) |
| **PUGLIA** | ASL LECCE – Centro profilassi dei viaggiatori | Lecce | Via Miglietta 5 | tel 0832 215332 | [medicinadeiviaggiatori@asl.le](mailto:medicinadeiviaggiatori@asl.lecce.it) [cce.it](mailto:medicinadeiviaggiatori@asl.lecce.it) |
| **PUGLIA** | ASL LECCE –  Medicina del turismo e delle migrazioni | Maglie | Via Sante Cezza 8 | tel 0836 425217 | [sisp.uo.casarano@asl.lecce.it](mailto:sisp.uo.casarano@asl.lecce.it) |
| **PUGLIA** | ASL TARANTO –  Ambulatorio vaccinale | Manduria | Via S. Gregorio Magno ang. Piazza della Pietà (ex Palazzo Scialpi) | tel 099 800123 | [diprev.sisp.manduria@asl.tara](mailto:diprev.sisp.manduria@asl.taranto.it) [nto.it](mailto:diprev.sisp.manduria@asl.taranto.it) |
| **PUGLIA** | USMAF SASN di  Puglia, Calabria e Basilicata - Unità Territoriale di  Manfredonia | Manfredonia | Viale Kennedy 3 | tel 06 5994 4727 | [usma.manfredonia@sanita.it](mailto:usma.manfredonia@sanita.it) |
| **PUGLIA** | ASL TARANTO –  Ambulatorio vaccinale | Martina Franca | Via Toniolo 6/R | tel 080 4835358 | [diprev.sisp.martinafranca@asl.](mailto:diprev.sisp.martinafranca@asl.taranto.it) [taranto.it](mailto:diprev.sisp.martinafranca@asl.taranto.it) |
| **PUGLIA** | ASL TARANTO –  Ambulatorio vaccinale | Massafra | Viale Magna Grecia  173 c/o  Ospedale Pagliari | tel 099 8850642 | [diprev.sisp.massafra@asl.taran](mailto:diprev.sisp.massafra@asl.taranto.it) [to.it](mailto:diprev.sisp.massafra@asl.taranto.it) |
| **PUGLIA** | ASL BRINDISI –  Ambulatorio vaccinale | Mesagne | Piazza Gioberti s.n.c | Tel 0831 739434 | [ufficiovaccinazioni.mesagne@](mailto:ufficiovaccinazioni.mesagne@asl.brindisi.it) [asl.brindisi.it](mailto:ufficiovaccinazioni.mesagne@asl.brindisi.it) |
| **PUGLIA** | ASL FOGGIA –  Ambulatorio vaccinale | San Severo | Via T. Masselli 28 | tel 0882 200587 | [sisp.direzionenord@aslfg.it](mailto:sisp.direzionenord@aslfg.it) |
| **PUGLIA** | USMAF SASN di  Puglia, Calabria e  Basilicata - Unità Territoriale di Taranto | TARANTO | Porto Mercantile | tel 06 5994 4726 | [usma.taranto@sanita.it](mailto:usma.taranto@sanita.it) |
| **PUGLIA** | ASL TARANTO –  Ambulatorio vaccinale | Taranto | Viale Magna Grecia 418 | tel 099 7786234 | [diprev.sisp.profilassi@asl.tara](mailto:diprev.sisp.profilassi@asl.taranto.it) [nto.it](mailto:diprev.sisp.profilassi@asl.taranto.it) |
| **SARDEGNA** | USMAF SASN  Campania e Sardegna – Unità Territoriale di  Cagliari | Cagliari | Banchina Riva di Ponente – Porto di Cagliari | tel 06 59944827  (Riva di Ponente) tel 06/59944828  (Porto Canale) | [usma.cagliari@sanita.it](mailto:usma.cagliari@sanita.it) |
| **SARDEGNA** | ASSL di Cagliari – Profilassi del viaggiatore internazionale | Cagliari | Via Is Guadazzonis c/o Stabilimento Binaghi ex Centro Trapianti 1° Piano | tel 070 6092917 |  |
| **SARDEGNA** | Azienda Usl 4 | Lanusei | Via Trento | tel 0782 470454 -  55 - 49 |  |
| **SARDEGNA** | ASL 3 | Nuoro | Via Trieste 80 | tel 0784 240847 |  |
| **SARDEGNA** | Centro vaccinale Distretto di Olbia - Medicina del viaggiatore c/o Polo sanitario San  Giovanni di Dio | Olbia | Via Borromini snc |  | [vaccinazione.perlavita@aslgall](mailto:vaccinazione.perlavita@aslgallura.it) [ura.it](mailto:vaccinazione.perlavita@aslgallura.it) |
| **SARDEGNA** | USMAF SASN  Campania e Sardegna – Unità Territoriale di PortoTorres | Porto Torres | Molo Teleferica Porto Civico Porto Torres via Campidano 7 Olbia | tel 06 5994 4824 | [usma.portotorres@sanita.it](mailto:usma.portotorres@sanita.it) |
| **SARDEGNA** | Azienda Usl 6 - Ambulatorio medicina dei viaggi | Sanluri | Via Bologna 13 - 09025 | tel 070-9359413 | [medicina.viaggi@aslmedioca](mailto:medicina.viaggi@aslmediocampidano.it) [mpidano.it](mailto:medicina.viaggi@aslmediocampidano.it) |

| **SARDEGNA** | ASL 1 - Igiene e sanità pubblica | Sassari | Via Rizzeddu 21/B - palazzina G | tel 079 2062875 -  078 2062869 | [prenotazionevaccini.sassari@a](mailto:prenotazionevaccini.sassari@aslsassari.it) [slsassari.it](mailto:prenotazionevaccini.sassari@aslsassari.it) |
| --- | --- | --- | --- | --- | --- |
| **SARDEGNA** | Asl 2 Medicina del  viaggiatore | Tempio Pausania | Via Demartis | tel 079 678345 |  |
| **SICILIA** | USMAF SASN di Sicilia  - Unità Territoriale di Augusta | Augusta | Via Darsena 23 | tel 06 5994 4761 | [usma.augusta@sanita.it](mailto:usma.augusta@sanita.it) |
| **SICILIA** | USMAF SASN di Sicilia  - Unità Territoriale di Catania | Catania | Via Dusmet snc - Porto di Catania- | tel 06 5994 4813 | [usma.catania@sanita.it](mailto:usma.catania@sanita.it) |
| **SICILIA** | Asl 3 Catania | Catania | Corso d'Italia 234 | tel 095 2545260 -  244 |  |
| **SICILIA** | USMAF SASN di Sicilia  - Unità Territoriale di Porto Empedocle- Presidio di Licata | Licata | Porto banchina Marianello - Licata ex mercato ittico (AG) | tel tel 06 509949445/9444 |  |
| **SICILIA** | USMAF SASN di Sicilia  - Unità Territoriale di Messina | Messina | Via Tommaso Cannizzaro 88 | tel 06 5994 4811 | [usma.messina@sanita.it-](about:blank) |
| **SICILIA** | USMAF SASN di Sicilia  - Unità Territoriale d**i** Palermo | Palermo | Molo Sammuzzo  snc Porto di Palermo | tel 0659944719 |  |
| **SICILIA** | USMAF SASN di Sicilia  - Unità Territoriale di Porto Empedocle | Porto Empedocle | Via Molo 60 | tel 06 5994 4760 | [usma.portoempedocle@sanita.i](mailto:usma.portoempedocle@sanita.it) [t](mailto:usma.portoempedocle@sanita.it) |
| **SICILIA** | Azienda Usl 7 | Ragusa | Via G. Di Vittorio 58D | tel 0932 234664 -  665 |  |
| **SICILIA** | USMAF SASN di Sicilia  - Unità Territoriale di Siracusa | Siracusa | Viale Montedoro 2 | tel 06 5994 4785 | [usma.siracusa@sanita.it](mailto:usma.siracusa@sanita.it) |
| **SICILIA** | USMAF SASN di Sicilia  - Unità Territoriale di Trapani | Trapani | Via Ammiraglio Staiti 23 | tel 06 5994 4755 | [usma.trapani@sanita.it](mailto:usma.trapani@sanita.it) |
| **TOSCANA** | USL Toscana Sud Est – Ambulatorio di medicina del viaggiatore | Abbadia San Salvatore (SI) | Via Serdini, 46 - Abbadia San Salvatore (SI) | CUP 0577/767676 |  |
| **TOSCANA** | USL Toscana Sud Est – Ambulatorio vaccinazioni adulti | Arezzo | Via Campo di Marte, 25 – 52100 Arezzo | 0575/254851 |  |
| **TOSCANA** | USL Toscana Sud Est – Ambulatorio vaccinazioni internazionali | Bibbiena (AR) | Via G. Vittorio, 1 – Bibbiena (AR) | 0575/568321 |  |
| **TOSCANA** | USL Toscana Nord Ovest - Centro per la medicina del viaggiatore  - Dipartimento della prevenzione | Capannori (LU) | Dipartimento della prevenzione P.zza A.Moro 55012  Capannori (LU) | 0583/449818 |  |
| **TOSCANA** | USL Toscana Nord Ovest - Centro di medicina dei viaggiatori  - Dipartimento della Prevenzione - Centro Polispecialistico “A. Sicari” | Carrara | Piazza Sacco e Vanzetti 1- Padiglione I – loc. Monterosso a Carrara (MS) – piano terreno | 0585/655848-  5235- 5848 |  |
| **TOSCANA** | USL Toscana Nord Ovest - Spazio Benessere srl | Casciavola (PI) | Via Novella, 5 Loc. Casciavola (PI) | 050 77 77 20 |  |
| **TOSCANA** | USL Toscana Nord Ovest – Ambulatorio di consulenza e profilassi per i viaggiatori internazionali | Cecina (LI) | Via Montanara, 4 (consultorio ospedale) 57123 Cecina (LI) | 0586/614305 |  |
| **TOSCANA** | USL Toscana Sud Est – Ambulatorio  vaccinazioni internazionali | Cortona (AR) | Via Capitini, 6 Loc. Camucia – Cortona (AR) | 0575/639960 |  |
| **TOSCANA** | USL Toscana Centro – Centro medicina dei viaggi | Empoli (FI) | Via dei Cappuccini, 79 - 50053 Empoli (FI) | 055/545454 |  |

| **TOSCANA** | USL Toscana Centro - Centro medicina dei viaggi e delle migrazioni | Firenze | Via di San Salvi, 12  - 50135 Firenze,  Palazzina N° 29 Igiene e Sanità Pubblica | CUP  appuntamenti 055/545454;  info 055/6933756  mercoledì e venerdì 8.30-  10.30 |  |
| --- | --- | --- | --- | --- | --- |
| **TOSCANA** | USL Toscana Centro - Humanray srl | Firenze | Via Quintino Sella, 62/R Firenze | 333/9767781 |  |
| **TOSCANA** | USL Toscana Sud Est - Ambulatorio viaggiatore internazionale – Centro  socio-sanitario | Follonica (GR) | Centro socio- sanitario V.le Europa  - 58022 Follonica  (GR) | 0566/59544 |  |
| **TOSCANA** | USL Toscana Nord Ovest - Centro per la medicina del viaggiatore | Gallicano (LU) | Dipartimento della Prevenzione – via IV Novembre10 55027  Gallicano (LU) |  |  |
| **TOSCANA** | USL Toscana Sud Est – Ambulatorio viaggiatore internazionale | Grosseto | Via Cimabue, 109  58100 Grosseto | 0564/485615-  485622 |  |
| **TOSCANA** | USMAF SASN di  Toscana, Emilia Romagna – Ufficio  Territoriale di Livorno | Livorno | Via Strozzi 1 | tel 06 5994 4799 | [usma.livorno@sanita.it](mailto:usma.livorno@sanita.it) |
| **TOSCANA** | USL Toscana Nord Ovest – Centro di consulenza e profilassi per i viaggiatori internazionali | Livorno | Borgo S. Jacopo, 59 57126 Livorno | 0586/223577 |  |
| **TOSCANA** | USL Toscana Nord Ovest - Centro per la medicina del viaggiatore | Lucca | EX Presidio Ospedaliero Campo di Marte padiglione "D" ambulatorio 8, 1  piano 55100 Lucca | 0583/449833-  970654 |  |
| **TOSCANA** | USL Toscana Nord Ovest - Centro di sanità solidale srl | Lucca | Via dei Macelli, 101 Lucca | 0583/327790 |  |
| **TOSCANA** | USL Toscana Centro Ambulatorio medicina del viaggiatore - Villa  Belvedere Ankuri Pucci | Massa e Cozzile (PT) | Via Primo Maggio 154 - Villa Belvedere Ankuri Pucci - 51010  Massa e Cozzile PT | 0572 942808 |  |
| **TOSCANA** | USL Toscana Sud Est – Ambulatorio del viaggiatore c/o Ospedali Riuniti della Val Di Chiana | Montepulciano di Nottola (SI) | Ospedali riuniti della Val di chiana Via Prov.5 - loc.  Montepulciano di Nottola | CUP 0577/767676 |  |
| **TOSCANA** | USL Toscana Sud Est – Ambulatorio per viaggiatori internazionali – c/o Ospedale del Valdarno S. Maria alla Gruccia | Montevarchi (AR) | Ospedale del Valdarno S.Maria alla Gruccia P.zza del Volontariato, 2  52025 - Montevarchi (AR) | 055/9106353 |  |
| **TOSCANA** | USL Toscana Sud Est - Ambulatorio viaggiatore internazionale c/o Presidio Ospedaliero S.  Giovanni di Dio | Orbetello (GR) | P.O.S.Giovanni di Dio Loc. Madonnella – 58015 Orbetello (GR) | 0564/869309 |  |
| **TOSCANA** | USL Toscana Nord Ovest - Ambulatorio medicina dei viaggi | Pietrasanta (LU) | Via Martiri di Sant'Anna, 12 -  55045 Pietrasanta  (LU) | 0584/6058849 -  50 |  |
| **TOSCANA** | USL Toscana Nord Ovest - Ambulatorio di consulenza e profilassi  per i viaggiatori internazionali | Piombino | Via Forlanini, 26  57025 Piombino | 0565/67534 |  |
| **TOSCANA** | USL Toscana Nord  Ovest - Ambulatorio medicina dei viaggi | Pisa | Galleria G.B.Gerace, 14 56124 Pisa | 050/954418 |  |

| **TOSCANA** | USMAF SASN di  Toscana, Emilia Romagna – Ufficio Territoriale di Pisa | Pisa | Piazzale Corradino D’Ascanio 1 Aereoporto G. Galilei Palazzina A  – scala B 1° Piano | tel 06 5994 4781 | [usma.pisa@sanita.it](mailto:usma.pisa@sanita.it) |
| --- | --- | --- | --- | --- | --- |
| **TOSCANA** | USL Toscana Centro - Ambulatorio medicina del viaggiatore | Pistoia | Viale Matteotti 19 Pistoia | CALL CENTER CUP 840.003.003 da  telefono fisso, 848.800.709;  oppure 199  175955 da rete mobile |  |
| **TOSCANA** | USL Toscana Sud Est - Ambulatorio del viaggiatore | Poggibonsi (SI) | Via della Costituzione, - 53036 Poggibonsi (SI) | CUP 0577/767676 |  |
| **TOSCANA** | USL Toscana Nord Ovest - Ambulatorio vaccinazione adulti e  viaggiatori | Pontedera | Via E.Mattei, 2  56025 Pontedera | 0587/273436-43-  098758 |  |
| **TOSCANA** | USL Toscana Nord Ovest - Ambulatorio di consulenza e profilassi per i viaggiatori  internazionali | Portoferraio (LI) | Largo Torchiana (ex Loc. S. Rocco) 57037 Portoferraio (LI) | 0565/926818 |  |
| **TOSCANA** | USL Toscana Centro – Ambulatorio per la medicina dei viaggi – Igiene e Sanità Pubblica | Prato | Igiene e Sanità Pubblica - Via Lavarone n. 3/5 59100 Prato | CUP  appuntamenti 055 545454;  info 0574/805341 |  |
| **TOSCANA** | USL Toscana Sud Est - Ambulatorio vaccinazioni adulti e  viaggiatori | Sansepolcro (AR) | Via Santi di Tito, 24 52037 – Sansepolcro (AR) | 0575/757869-  757879-757866 |  |
| **TOSCANA** | USL Toscana Sud Est – Ambulatorio di medicina del viaggiatore | Siena | Strada del Ruffolo - 53100 Siena | Numero CUP 0577/767676 |  |
| **TOSCANA** | USL Toscana Nord Ovest - Ambulatorio medicina dei viaggi | Volterra (PI) | Borgo San Lazzaro, 5 - 56048 Volterra (PI) | 0588/91813 |  |
| **UMBRIA** | Azienda USL Umbria 2 | Foligno | Via Aspromonte 8, c/o Centro commerciale "Agorà" | tel 0742 321685 | [vaccinazioni.foligno@uslumbr](mailto:vaccinazioni.foligno@uslumbria2.it) [ia2.it](mailto:vaccinazioni.foligno@uslumbria2.it) |
| **UMBRIA** | Azienda USL Umbria 1 - Centro Medicina dei viaggiatori | Perugia | Via XIV Settembre 79 – Parco Santa Margherita –  Palazzina Zurli | tel 075 5412454 | [cmviaggiatori@uslumbria1.it](mailto:cmviaggiatori@uslumbria1.it) |
| **UMBRIA** | Azienda USL Umbria 2 | Spoleto | Via San Carlo 1 | tel 0743 210701 |  |
| **UMBRIA** | Azienda USL Umbria 2 - Medicina dei viaggiatori | Terni | Via Bramante 37 | tel 0744  2043401-  204338-204337-  204336 | [igiene.sanita.pubblica@uslumb](mailto:igiene.sanita.pubblica@uslumbria2.it) [ria2.it](mailto:igiene.sanita.pubblica@uslumbria2.it) |
| **VALLE D'AOSTA** | SC Igiene e Sanità Pubblica | Aosta | Via Saint Martin De Corleans 250 | tel 0165 546080 |  |
| **VENETO** | ULSS 8 Berica Servizio Igiene e Sanità Pubblica  - Ambulatorio Viaggiatori Internazionali Distretto  Ovest | Arzignano (VI) | Via Kennedy 2 - 36071 | tel 0444-708673 | [igienepubblica.arzignano@auls](mailto:igienepubblica.arzignano@aulss8.veneto.it) [s8.veneto.it](mailto:igienepubblica.arzignano@aulss8.veneto.it) |
| **VENETO** | ULSS 7 Pedemontana Ambulatorio Viaggiatori  Internazionali | Bassano del Grappa (VI) | Via Cereria 15 - 36016 | tel 0424 885500 | [asv.sisp@aulss7.veneto.it](mailto:asv.sisp@aulss7.veneto.it) |
| **VENETO** | ULSS 1 Dolomiti - Servizio Igiene e Sanità Pubblica | Belluno | Viale Europa 22 - 32100 | tel 0437 514525 | [vaccinazioni.bl@aulss1.veneto](mailto:vaccinazioni.bl@aulss1.veneto.it)  [.it](mailto:vaccinazioni.bl@aulss1.veneto.it) |
| **VENETO** | ULSS 6 Euganea Servizio Igiene e Sanità Pubblica | Camposampiero (PD) | Via Cao del Mondo 1- (Centro De  Rossignoli) - 35012 | tel 049-9822202 | [ambulatorio_sisp@aulss6.vene](mailto:ambulatorio_sisp@aulss6.veneto.it) [to.it](mailto:ambulatorio_sisp@aulss6.veneto.it) |

| **VENETO** | ULSS 3 SERENISSIMA  Servizio Igiene e Sanità Pubblica - Sede Chioggia | Chioggia | Borgo S. Giovanni 1183/C - 30015 | tel 800 938811 | [sisp.vaccinazioni@aulss3.vene](mailto:sisp.vaccinazioni@aulss3.veneto.it) [to.it](mailto:sisp.vaccinazioni@aulss3.veneto.it) |
| --- | --- | --- | --- | --- | --- |
| **VENETO** | ULSS 6 Euganea Servizio Igiene e Sanità Pubblica | Cittadella (PD) | Via Pilastroni (Centro Pontarollo) - 35013 | tel 049-9424433 | [ambulatorio_sisp@aulss6.vene](mailto:ambulatorio_sisp@aulss6.veneto.it) [to.it](mailto:ambulatorio_sisp@aulss6.veneto.it) |
| **VENETO** | ULSS 2 Marca Trevigiana – Servizio igiene e sanità pubblica –  Sede territoriale di Conegliano | Conegliano (TV) | Via Galvani 4 - 31015 | tel 0438 663928 | [vaccinazioni.pieve@aulss2.ven](mailto:vaccinazioni.pieve@aulss2.veneto.it) [eto.it](mailto:vaccinazioni.pieve@aulss2.veneto.it) |
| **VENETO** | ULSS 3 SERENISSIMA  Servizio Igiene e Sanità Pubblica - Sede Dolo | Dolo (VE) | Riviera XXIX Aprile 2 - 30031 | tel 800 938811 | [sisp.vaccinazioni@aulss3.vene](mailto:sisp.vaccinazioni@aulss3.veneto.it) [to.it](mailto:sisp.vaccinazioni@aulss3.veneto.it) |
| **VENETO** | ULSS 6 Euganea Servizio Igiene e Sanità Pubblica | Este (PD) | Via Francesconi 2 - 35042 | tel 0429 618555 | [ambsisp.este@aulss6.veneto.it](mailto:ambsisp.este@aulss6.veneto.it)  , [sisp.este@aulss6.veneto.it](mailto:sisp.este@aulss6.veneto.it) |
| **VENETO** | ULSS 1 Dolomiti – Servizio Igiene e Sanità Pubblica | Feltre (BL) | Via Bagnols sur Ceze 30 - 32032, c/o Palazzina Fusaro | tel 0439 883417 | [vaccinazioni.fe@aulss1.veneto](mailto:vaccinazioni.fe@aulss1.veneto.it)  [.it](mailto:vaccinazioni.fe@aulss1.veneto.it) |
| **VENETO** | ULSS 4 Veneto Orientale Servizio Igiene e Sanità Pubblica | Jesolo | Via Levantina 104 - 30016 | tel 0421-228163 | [vaccinazioni@aulss4.veneto.it](mailto:vaccinazioni@aulss4.veneto.it) |
| **VENETO** | ULSS 9 Scaligera  Ambulatorio Viaggiatori Internazionali | Legnago (VR) | Via Gianella 1 -  37045, c/o Ospedale Mater Salutis | tel 0442- 622642/2955 |  |
| **VENETO** | ULSS 3 SERENISSIMA  Servizio Igiene e Sanità Pubblica - Sede Terraferma Veneziana | Mestre | P.le San Lorenzo Giustiniani 11/D - 30174 | tel 800 938811 | [sisp.vaccinazioni@aulss3.vene](mailto:sisp.vaccinazioni@aulss3.veneto.it) [to.it](mailto:sisp.vaccinazioni@aulss3.veneto.it) |
| **VENETO** | ULSS 2 Marca Trevigiana – Servizio igiene e sanità pubblica –  Sede territoriale di Montebelluna | Montebelluna (TV) | Via Dante Alighieri  12 - 31044 | tel 0423-614714-  0423-611611 | [sisp.asolo@aulss2.veneto.it](mailto:sisp.asolo@aulss2.veneto.it) |
| **VENETO** | Centro Malattie Tropicali Ospedale Sacro Cuore di Negrar | Negrar (VR) | Via Sempreboni, 5 - 37024 | tel. 045 6013324  - 4293 |  |
| **VENETO** | ULSS 2 Marca Trevigiana – Servizio di Igiene e Sanità Pubblica  – Sede territoriale di Oderzo | Oderzo | Via D. Manin 46 - 31046 | tel 0422 715601 | [sispmalattieinfettive@aulss2.v](mailto:sispmalattieinfettive@aulss2.veneto.it) [eneto.it](mailto:sispmalattieinfettive@aulss2.veneto.it) |
| **VENETO** | ULSS 6 Euganea Servizio Igiene e Sanità Pubblica | Padova | Via Ospedale 22 - 35121 | tel CUP da rete fissa 840 000 664 e da rete mobile  049 8239511 | [sisp.vaccinazioni@aulss6.vene](mailto:sisp.vaccinazioni@aulss6.veneto.it) [to.it](mailto:sisp.vaccinazioni@aulss6.veneto.it) |
| **VENETO** | ULSS 6 Euganea Distretto 3 sede di Piove di Sacco | Piove di Sacco (PD) | Via San Rocco 8 - 35028 | tel CUP da rete fissa 840 000 664 e da rete mobile 049 8239511 | [sisp.vaccinazioni@aulss6.vene](mailto:sisp.vaccinazioni@aulss6.veneto.it) [to.it](mailto:sisp.vaccinazioni@aulss6.veneto.it) |
| **VENETO** | ULSS 4 Veneto  Orientale Servizio Igiene e Sanità Pubblica | Portogruaro (VE) | Via Zappetti 23 - 30026 | tel 0421-228163 | [vaccinazioni@aulss4.veneto.it](mailto:vaccinazioni@aulss4.veneto.it) |
| **VENETO** | ULSS 5 Polesana Servizio Igiene e Sanità Pubblica | Rovigo | Viale Tre Martiri 89  - 45100 | 0425 393745 | [vaccinazionirovigo@aulss5.ve](mailto:vaccinazionirovigo@aulss5.veneto.it) [neto.it](mailto:vaccinazionirovigo@aulss5.veneto.it) |
| **VENETO** | ULSS 4 Veneto Orientale Servizio Igiene  e Sanità Pubblica | S. Donà di Piave (VE) | Via Trento 17 - 30027 | tel 0421-228163 | [vaccinazioni@aulss4.veneto.it](mailto:vaccinazioni@aulss4.veneto.it) |
| **VENETO** | ULSS 7 Pedemontana Ambulatorio Viaggiatori Internazionali | Thiene (VI) | Via Boldrini 1 - 36016 | tel0445-389393 | [vacinf@aulss7.veneto.it](mailto:vacinf@aulss7.veneto.it) |
| **VENETO** | ULSS 2 Marca Trevigiana – Servizio di Igiene e Sanità Pubblica  – Sede territoriale di Treviso | Treviso | Via Castellana 2 - 31100 | tel 0422323811-  12 | [sispmalattieinfettive@aulss2.v](mailto:sispmalattieinfettive@aulss2.veneto.it) [eneto.it](mailto:sispmalattieinfettive@aulss2.veneto.it) |

| **VENETO** | USMAF SASN di  Veneto, Friuli Venezia Giulia e Trentino Alto Adige - VENEZIA | Venezia | Fondamenta Zattere, 1416 – Dorsoduro  1416 | tel 041 5102377;  tel 06 59944836  (sede di Venezia) | [usma.venezia@sanita.it](mailto:usma.venezia@sanita.it) |
| --- | --- | --- | --- | --- | --- |
| **VENETO** | ULSS 3 SERENISSIMA  Servizio Igiene e Sanità Pubblica - Sede Venezia Centro Storico | Venezia | Dorsoduro 1454 - ex Ospedale G. B. Giustinian -30123 | tel 800 938811 | [sisp.vaccinazioni@aulss3.vene](mailto:sisp.vaccinazioni@aulss3.veneto.it) [to.it](mailto:sisp.vaccinazioni@aulss3.veneto.it) |
| **VENETO** | ULSS 9 Scaligera Servizio Igiene e Sanità Pubblica | Verona | Via Salvo D'acquisto 7- 37122 | tel 045-8075047  (Per prenotazioni 045-8075918) | [viaggiatori.internazionali@aul](mailto:viaggiatori.internazionali@aulss9.veneto.it) [ss9.veneto.it](mailto:viaggiatori.internazionali@aulss9.veneto.it) |
| **VENETO** | ULSS 8 Berica Servizio Igiene e Sanità Pubblica  - Ambulatorio Viaggiatori Internazionali Distretto  Est | Vicenza | Via IV Novembre 46  - 36100 | tel 0444-752218 | [sisp.vicenza@aulss8.veneto.it](mailto:sisp.vicenza@aulss8.veneto.it) |
| **VENETO** | ULSS 2 Marca Trevigiana – Servizio igiene e sanità pubblica - Sede territoriale di  Vittorio Veneto | Vittorio Veneto (TV) | Piazza Foro Boario 9  -31029 | tel 0438 663928 | [vaccinazioni.pieve@aulss2.ven](mailto:vaccinazioni.pieve@aulss2.veneto.it) [eto.it](mailto:vaccinazioni.pieve@aulss2.veneto.it) |
