# Supplementary material for: A decalogue for personalized travel health assistance with AI-driven chatbots
Source: J Travel Med. 2024 Feb 12;31(4):taae026. doi: 10.1093/jtm/taae026 (PMC11149716; doi:10.1093/jtm/taae026)
Supplement: S4_JTM_Baglivo_taae026 [file s4_jtm_baglivo_taae026.docx]

**Supplementary S4**

Link of the chat examples. Each chat is numbered. There is also a short description of the chat and the language used.

| **Chat n°** | **Chat Link** | **Brief description** | **Language** |
| --- | --- | --- | --- |
| Chat_1 | <https://chat.openai.com/share/6b99ceab-952f-4a5a-9afb-f9073ce3f604> | 45 years old diabetic traveler going to India living in Genoa (Italy). | English |
| Chat_2 | <https://chat.openai.com/share/f2cd5c59-73ab-4830-b0fc-2fcff9f8b604> | Traveler feeling unhealthy during a trip in Morocco. | English |
| Chat_3 | <https://chat.openai.com/share/83419f6c-1f76-4022-a923-e4581965a4fd> | Italian traveler, currently in Cyprus, seeking advice for his next trip to Senegal. | Italian |
| Chat_4 | <https://chat.openai.com/share/e5e94fd2-d81b-429c-b1af-035b344864f7> | A traveler planning a trip to Guatemala is worried about dialysis. | Italian |
| Chat_5 | <https://chat.openai.com/share/5f960f29-83a0-4c9e-8c0c-7befba71f1dc> | 85 years old cardiopathic going to Japan with unknown vaccination history | Italian |
| Chat_6 | <https://chat.openai.com/share/bb1f4c82-485e-46e1-baf1-e173833f6881> | Dengue vaccination in Turkey? | English |
| Chat_7 | <https://chat.openai.com/share/dc0b7bec-2808-4e6e-a456-d84ff70daf9b> | Health Insurance in the UK. | English |
| Chat_8 | <https://chat.openai.com/share/2f5c1840-45b0-4a5d-bc35-7a19044d42ca> | Symptoms post Tanzania Safari. | English |
| Chat_9 | <https://chat.openai.com/share/58f62d36-6420-4a62-aa6c-2c42ee205fde> | Hematuria before traveling to Berlin. | English |
| Chat_10 | <https://chat.openai.com/share/3b6714eb-77ce-4956-8b4f-29c085b4f697> | Help for acute gout in Milan | French |
| Chat_11 | <https://chat.openai.com/share/7bb97b0c-d03f-4c55-95f3-88c7354487ca> | Rabies vaccination between going to Botswana | English |
| Chat_12 | <https://chat.openai.com/share/c47f8fee-bcd7-4044-9bdd-c906c9a5dfd6> | Managing Medication Across Timezones | English |
| Chat_13 | <https://chat.openai.com/share/17c54a3c-36c3-4c9b-8937-2d8d36babfd2> | Traveling with Lupus to South Africa | English |
| Chat_14 | <https://chat.openai.com/share/44bbd44e-c6de-4587-8065-50a9393215c9> | Malaria symptoms after a trip to Indonesia | Italian |
| Chat_15 | <https://chat.openai.com/share/7e893bae-88e9-4d2e-91c1-67ad3ec04c79> | Traveling with Flu Symptoms | English |
| Chat_16 | <https://chat.openai.com/share/07c41742-3807-4c14-8cf9-f617c39c556c> | Traveling to Huaraz (Perù) | Spanish |
| Chat_17 | <https://chat.openai.com/share/a678e47e-e5ff-49b0-ba0b-a022bccc331c> | Travel to Venezuela of a traveler with heart disease and no passport | Spanish |
| Chat_18 | <https://chat.openai.com/share/6afcaa3c-bfce-45f3-886b-9b723951d9ae> | 33 years old Chinese VFR with no vaccinations who take cortisone | Chinese |
